# NLRX1 Facilitates *Histoplasma capsulatum*-Induced LC3-Associated Phagocytosis for Cytokine Production in Macrophages

**DOI:** 10.3389/fimmu.2018.02761

**Published:** 2018-12-03

**Authors:** Juin-Hua Huang, Chu-Yu Liu, Sheng-Yang Wu, Wen-Yu Chen, Tzu-Hsuan Chang, Hung-Wei Kan, Sung-Tsang Hsieh, Jenny P.-Y. Ting, Betty A. Wu-Hsieh

**Affiliations:** ^1^Graduate Institute of Immunology, National Taiwan University College of Medicine, Taipei, Taiwan; ^2^Department of Anatomy and Cell Biology, National Taiwan University College of Medicine, Taipei, Taiwan; ^3^Departments of Genetics, Microbiology and Immunology, Lineberger Comprehensive Cancer Center, Center for Translational Immunology, University of North Carolina, Chapel Hill, NC, United States

**Keywords:** LC3-associated phagocytosis, *Histoplasma capsulatum*, NLRX1, macrophage, non-canonical autophagy, cytokine response, innate anti-fungal immunity

## Abstract

LC3-associated phagocytosis (LAP) is an emerging non-canonical autophagy process that bridges signaling from pattern-recognition receptors (PRRs) to autophagic machinery. LAP formation results in incorporation of lipidated LC3 into phagosomal membrane (termed LAPosome). Increasing evidence reveals that LAP functions as an innate defense mechanism against fungal pathogens. However, the molecular mechanism involved and the consequence of LAP in regulating anti-fungal immune response remain largely unexplored. Here we show that *Histoplasma capsulatum* is taken into LAPosome upon phagocytosis by macrophages. Interaction of *H. capsulatum* with Dectin-1 activates Syk and triggers subsequent NADPH oxidase-mediated reactive oxygen species (ROS) response that is involved in LAP induction. Inhibiting LAP induction by silencing LC3α/β or treatment with ROS inhibitor impairs the activation of MAPKs-AP-1 pathway, thereby reduces macrophage proinflammatory cytokine response to *H. capsulatum*. Additionally, we unravel the importance of NLRX1 in fungus-induced LAP. NLRX1 facilitates LAP by interacting with TUFM which associates with autophagic proteins ATG5-ATG12 for LAPosome formation. Macrophages from *Nlrx1*^−/−^ mice or TUFM-silenced cells exhibit reduced LAP induction and LAP-mediated MAPKs-AP-1 activation for cytokine response to *H. capsulatum*. Furthermore, inhibiting ROS production in *Nlrx1*^−/−^ macrophages almost completely abolishes *H. capsulatum*-induced LC3 conversion, indicating that both Dectin-1/Syk/ROS-dependent pathway and NLRX1-TUFM complex-dependent pathway collaboratively contribute to LAP induction. Our findings reveal new pathways underlying LAP induction by *H. capsulatum* for macrophage cytokine response.

## Introduction

*Histoplasma capsulatum* is a pathogenic dimorphic fungus that can cause flu-like respiratory illness in humans. The infection can become life-threatening when it disseminates from lungs to other organs (1). Cases of histoplasmosis are reported worldwide (2, 3). The incidence of progressive disseminated histoplasmosis may continue to rise due to increased international travel and extensive use of immunosuppressive medications. Infection of *H. capsulatum* is initiated by inhalation of microconidia or fragments of hyphae. The hyphal forms then undergo a morphological transform to budding yeasts, which are taken up by macrophages (4). Engulfed *H. capsulatum* interferes with the acidification of phagolysosome and subsequently survives and replicates within macrophages (5, 6). Recognition of *H. capsulatum* by macrophage through CR3 and Dectin-1 triggers TNF and IL-6 production that orchestrates adaptive immune response against the infection (7). Mice defective in both CR3 and Dectin-1 are impaired in TNF and IL-6 production, which results in reduced Th1 and Th17 responses and heightened susceptibility to histoplasmosis (7). Macrophage also serves as an antigen donor cell to deliver *H. capsulatum* antigen to dendritic cells (DCs) for cross-presentation and functions as an effector cell to kill the intracellular yeasts when activated by IFN-γ, IL-17A, TNF, and GM-CSF (8–12). Given the multiple roles of macrophage in host defense against *H. capsulatum*, a better understanding of its interaction with the fungus is crucial for the development of new therapeutic strategies against histoplasmosis.

Macroautophagy (herein referred to as autophagy) pathway is involved in a wide range of innate and adaptive immune responses. It is known to regulate inflammatory signaling, antigen processing and presentation, and clearance of pathogens (13, 14). An emerging non-canonical autophagic process, LC3-associated phagocytosis (LAP), links cell surface receptor signaling to autophagic machinery. In LAP, lipidated LC3 (LC3-II) is recruited and incorporated into phagosomal membrane (termed LAPosome) that mediates phagosome maturation (15, 16). LAP pathway can serve as an innate defense mechanism against invading microorganisms including bacteria, fungi, and parasites (17, 18). In the context of fungal infection, *Candida albicans, Cryptococcus neoformans*, and *Aspergillus fumigatus* have been shown to be targeted by LAP in macrophages (19–23). Induction of LAP by *C. albicans* and *A. fumigatus* is triggered by Dectin-1/Syk signaling and requires NADPH oxidase-derived ROS response (19, 21– 23). It is reported that LAP facilitates the killing of fungi and plays a crucial role in controlling infections (20–24). Yet the role of LAP in anti-fungal immunity against *H. capsulatum* has never been studied. In addition to the direct effect on fungicidal functions, LAP impairment alters macrophage anti-fungal cytokine response (20, 22), indicating the involvement of LAP in inflammation modulation. Further studies are required to unravel how LAP affects the signaling pathway leading to cytokine production.

NLRX1 (also known as CLR11.3 and NOD9) is ubiquitously expressed in a variety of cell types and is the only NLR member that primarily localizes to the mitochondria (25, 26). NLRX1 is reported to be involved in regulation of several cellular functions, including innate inflammatory response, cell apoptosis, autophagy, and mitochondrial activity (25–31). Through association with different partners, NLRX1 acts as a negative regulator to inhibit TLR, MAVS, and STING pathways, and as a positive regulator to facilitate autophagy in response to viral infection (25, 27–29, 32). Mouse embryonic fibroblasts and primary peritoneal macrophages deficient in NLRX1 fail to induce LC3 conversion after infection with vesicular stomatitis virus (VSV) (29). Mechanistically, NLRX1 forms a complex with a mitochondrial protein Tu translation elongation factor (TUFM) which interacts with ATG5-ATG12 and ATG16L1, thereby promotes autophagy induction (29). Since ATG5-ATG12 and ATG16L1 are required for both canonical autophagy and LAP pathways, it is plausible that NLRX1 is involved in the LAP pathway and regulates host response against fungal infections.

In this study, we demonstrated the formation of LAP in *H. capsulatum*-infected macrophages. We discovered that signaling downstream of Dectin-1 facilitated NADPH oxidase-derived ROS response which was required for LAP induction by *H. capsulatum*. Through a mechanism independent of ROS, NLRX1 promotes fungus-induced LAP through its association with TUFM that interacted with ATG5-ATG12 conjugate for LAPosome formation. These two separate pathways collaboratively contributed to LAP induction. In addition, LAP formation promoted macrophage inflammatory cytokine response to *H. capsulatum* by enhancing MAPKs-AP-1 pathway. Here we revealed for the first time the role of *H. capsulatum*-induced LAP in macrophage cytokine response and the molecular mechanism of LAPosome formation. Our study also provides a link between NLRX1 and LAP in the context of host-fungus interactions.

## Materials and methods

### Fungus

*Histoplasma capsulatum* strain 505 yeast cells were cultured at 37°C on brain heart infusion (BHI) agar (BD Biosciences) supplemented with 1 mg/ml cysteine (Sigma), 20 mg/ml dextrose, and 10% heat-inactivated fetal bovine serum (FBS; Biological Industries). Yeast suspensions were freshly prepared in RPMI 1640 medium (Gibco) for each experiment. Heat-killed yeasts were prepared by treatment at 65°C for 2 h.

### Mice and cells

Wild-type C56BL/6 mice (The Jackson Laboratories; Stock number: 000664), *Itgam*^−/−^ (The Jackson Laboratories; Stock number: 003991), *Ncf1*^−/−^ (The Jackson Laboratories; Stock number: 027331), *Nlrx1*^−/−^ (27), *Clec7a*^−/−^ (33), *Clec4n*^−/−^ (34), and *Syk*^−/+^ (35) mice were bred and maintained in the National Laboratory Animal Center (NLAC, Taiwan) or in the National Taiwan University College of Medicine Laboratory Animal Center (NTU CMLAC) under specific pathogen-free (SPF) conditions. Mice at 6–12 weeks of age were used in all of the experiments.

Peritoneal macrophages were harvested by peritoneal lavage from mice at 4 days after peritoneal injection of 1 ml of 3% thioglycollate medium (Sigma-Aldrich). Peritoneal cells were allowed to adhere in the wells overnight, and the adherent cells were used in the experiments (>95% F4/80^+^). To obtain primary macrophages deficient in Syk, *Syk*^−/−^ embryos (E13.5-E15.5) were separated from *Syk*^+/+^ and *Syk*^+/−^ embryos by their exhibition of petechiae and verified by genotyping (36). Single-cell suspensions from fetal liver tissues were cultured in DMEM medium (Gibco) containing 20% L929 cell conditioned medium for 5 days. Over 95% of the adherent cells were F4/80^+^ which were identified as fetal liver-derived macrophages (FLDMs).

### Reagents and antibodies

Syk inhibitors SykI and BAY 61-3606, JNK inhibitor SP600125, ERK inhibitor U0126, and p38 inhibitor SB203580 were obtained from Calbiochem-Merck. Laminarin and zymosan were purchased from InvivoGen. Peptide inhibitor for NADPH oxidase assembly, gp91ds-tat, was purchased from AnaSpec. Diphenyleneiodonium chloride (DPI) and Mito-TEMPO were obtained from Sigma-Aldrich. LysoTracker Red was obtained from Life Technologies.

Antibodies against LC3, Rubicon, NLRX1, phospho (p)-p40-phox (Thr154), p-JNK (Thr183/Tyr185), p-ERK1/2 (Thr202/Tyr204), p-p38 (Thr180/Tyr182), p-c-Fos (Ser32), p-IKKα/β (Ser176/180), p-IκBα (Ser32), IκBα, and p-NFκBp65 (Ser536) were purchased from Cell Signaling. Antibodies against p-Syk (Tyr525), p-c-Jun (Ser63), and TUFM were obtained from Abcam. Anti-Syk, anti-ATG5, anti-β-actin, HRP-conjugated anti-rabbit IgG, and Rabbit IgG isotype control were purchased from GeneTex Inc. Receptor blocking antibodies against Dectin-1 (clone 2A11), Dectin-2 (clone D2.11E4), and CR3 (clone 5C6) were purchased from Bio-Rad (formerly AbD Serotec) and TLR2 (clone 6C2) was from eBioscience.

### Western blotting

Cell lysates were extracted by using PhosphoSafe Extraction Reagent cell lysis buffer (Novagen) following manufacturer's instructions. Cell lysates were subjected to electrophoresis at 7, 10, or 12.5% sodium dodecyl sulfate poly-acrylamide gel (SDS-PAGE) and transferred to PVDF membrane (GE Healthcare). The membrane was blocked with 5% skim milk (Sigma-Aldrich) and incubated in buffer containing primary antibody against molecule of interest followed by HRP-conjugated secondary antibodies. The blot was developed by chemiluminescence using ECL solution (GeneTex Inc., Merck Millipore, PerkinElmer Life Science, and GE Healthcare). Protein expression was quantified by densitometric analysis with ImageJ software (NIH, USA).

### Immunofluorescence staining and confocal microscopy

Macrophages were seeded and let adhere on cover slide overnight. After stimulation with *H. capsulatum* (MOI = 5), cells were fixed with 3% paraformaldehyde and permeabilized with 0.05% Triton X-100. Cells were then blocked with PBS containing 5% heat-inactivated FBS and stained with rabbit anti-LC3B (Cell signaling), biotin-labeled rabbit anti-NLRX1 (Proteintech), rabbit anti-TUFM (Abcam), and rat anti-LAMP2 (BioLegend) antibodies followed by Alexa Flour 488-conjugated anti-rabbit IgG, Alexa Flour 594-conjugated anti-biotin, and Alexa Flour 488-conjugated anti-rat IgG secondary antibodies (Jackson ImmunoResearch). F-actin was stained with CytoPainter Phalloidin-iFluor 647 (Abcam). Cell nuclei were stained with Hoechst 33258 (Thermo Fisher). The images were acquired with a Zeiss Axiovert 100VT confocal microscope (Carl Zeiss Inc.) and analyzed by LSM Image Browser (Carl Zeiss Inc.) and ImageJ software (NIH, USA).

### Transmission electron microscopy (TEM)

To analyze the membrane structure of a phagosome containing one single yeast, macrophages were stimulated with *H. capsulatum* at a low yeast-to-macrophage ratio (MOI = 2) for 30 min. After stimulation, cells were washed by Dulbecco's Phosphate-Buffered Saline (DPBS) and detached by using Accutase Cell Detachment Solution (BD Biosciences). Cells for TEM were processed as described previously (37). In brief, cells were pelleted, fixed in 5% glutaraldehyde, post-fixed with 2% osmic acid, dehydrated, and embedded in Epon 812 resin (Polysciences). Ultrathin sections (70 nm) were cut and stained with uranyl acetate and lead citrate before observation under a transmission electron microscope (H7100, Hitachi).

### ROS detection

Macrophages were stained with 5 μM CM-H_2_DCFDA (Life Technologies) in phenol red-free HBSS (Gibco) for 30 min. After replenishment with phenol red-free RPMI 1640 medium (Gibco), cells were let rest for 30 min. Due to the insensitivity of the assay, macrophages were stimulated with *H. capsulatum* yeasts at a high yeast-to-macrophage ratio (MOI = 10) for different periods of time. After stimulation, cells were washed with DPBS and detached by gently scrapping with a rubber policeman. Oxidative DCF was analyzed by flow cytometry.

### Phagocytosis assay

Macrophages were cooled on ice for 20 min before addition of FITC-labeled *H. capsulatum* at a yeast-to-macrophage ratio of 10/1 (38). FITC-labeled *H. capsulatum* was prepared freshly as previous described (38). Cells were then left on ice for another 60 min, followed by culturing in CO_2_ incubator at 37°C to allow phagocytosis. After incubation for 60 min, cells were washed by warm HBSS and the unphagocytosed FITC-labeled yeasts were quenched by treatment with trypan blue (1 mg/ml). After wash with warm DPBS, cells were detached by treatment with 5 mM EDTA and then fixed in 1% paraformaldehyde. The phagocytosis rate was determined by flow cytometry.

### Small interfering RNA (siRNA) transfection of macrophages

Macrophages were transfected with control siRNA or siRNA pools targeting Rubicon, LC3α/β or TUFM (Santa Cruz Biotechnology) using *Trans*IT-TKO Transfection Reagent (Mirus Biology LCC) according to the manufacturer's instruction. In brief, macrophages were cultured in RPMI 1640 medium containing 10% heat-inactivated FBS and allowed to adhere overnight. The siRNA transfection complex was formed by mixing the *Trans*IT-TKO Reagent with siRNA (25, 50, or 100 nM) in serum-free Opti-MEM medium (Gibco) at room temperature for 25 min and added dropwise to the cells. To assess knockdown efficiency, cells were collected at 30 and 72 h after incubation and subjected to real-time qPCR and Western blotting analysis, respectively. Medium was replaced prior to stimulation.

### Cytokine assay

Macrophages were stimulated with or without *H. capsulatum* yeasts (MOI = 5). At 18 h after stimulation, cell-free culture supernatants were harvested. The concentrations of TNF, IL-6 and IL-1β in the supernatants were quantified by enzyme-linked immunosorbent assay (ELISA) Ready-Set-Go kits (eBioscience) following the manufacturer's instruction.

### Co-immunoprecipitation (Co-IP)

Macrophages were stimulated with or without *H. capsulatum* (MOI = 5) for 1 h before lysis with non-denaturing lysis buffer [20 mM Tris HCl pH 8, 137 mM NaCl, 1% Nonidet P-40 (NP-40), 2 mM EDTA] supplemented with protease inhibitor cocktail (Sigma-Aldrich) and 25 mM N-ethylmaleimide (NEM). Whole cell lysates were incubated with antibodies against NLRX1 (Cell signaling), TUFM (Abcam), or rabbit IgG isotype control (GeneTex) at 4°C overnight followed by mixing with Protein A agarose beads (Merck Millipore) at 4°C for another 4 h. Lysate beads mixture was washed with IP washing buffer (0.1% NP-40 in PBS). The immunoprecipitates were eluted by being boiled in PhosphoSafe Extraction Reagent cell lysis buffer (Novagen) containing 1 × SDS-PAGE Loading Buffer (BIOTOOLS) and subjected to Western blotting.

### Replication time of intracellular *H. capsulatum*

The experiment followed a protocol that has been published previously (10). Briefly, macrophages were cultured with *H. capsulatum* yeasts (MOI = 2) for 1 h followed by wash to rid unenglufed yeasts. Cells were lysed by hypotonic shock with ddH_2_O (pH = 11) immediately (0 h) or after 18 h of incubation, and the number of viable intracellular yeast cells was counted by trypan blue exclusion. Replication time (h) = incubation interval/number of divisions; and number of divisions = log 2 (N_t_/N_0_), where N_t_ is the mean number of yeasts/infected macrophage at the end of incubation (18 h), and N_0_ is the mean number of yeasts/infected macrophage at time zero (0 h).

### Statistics

The difference between two groups was assessed by two-tailed *t*-test. The comparisons among multiple groups were analyzed by ANOVA followed by Bonferroni's multiple comparisons *post-hoc* test or Tukey *post-hoc* analysis. Statistical analysis was performed using GraphPad Prism software (GraphPad Software). Differences were considered significant at a *P*-value of ≤ 0.05.

### Ethics statement

All animal experiments were performed in accordance with the Guidebook for the Care and Use of Laboratory Animals, Eighth Edition, 2015, published by The Chinese-Taipei Society of Laboratory Animal Sciences. The animal protocol was approved by the Institutional Animal Care and Use Committee (IACUC, Permit number: 20140533) of National Taiwan University College of Medicine.

## Results

### *H. capsulatum* induces LAP in macrophages

**Figure 1:**
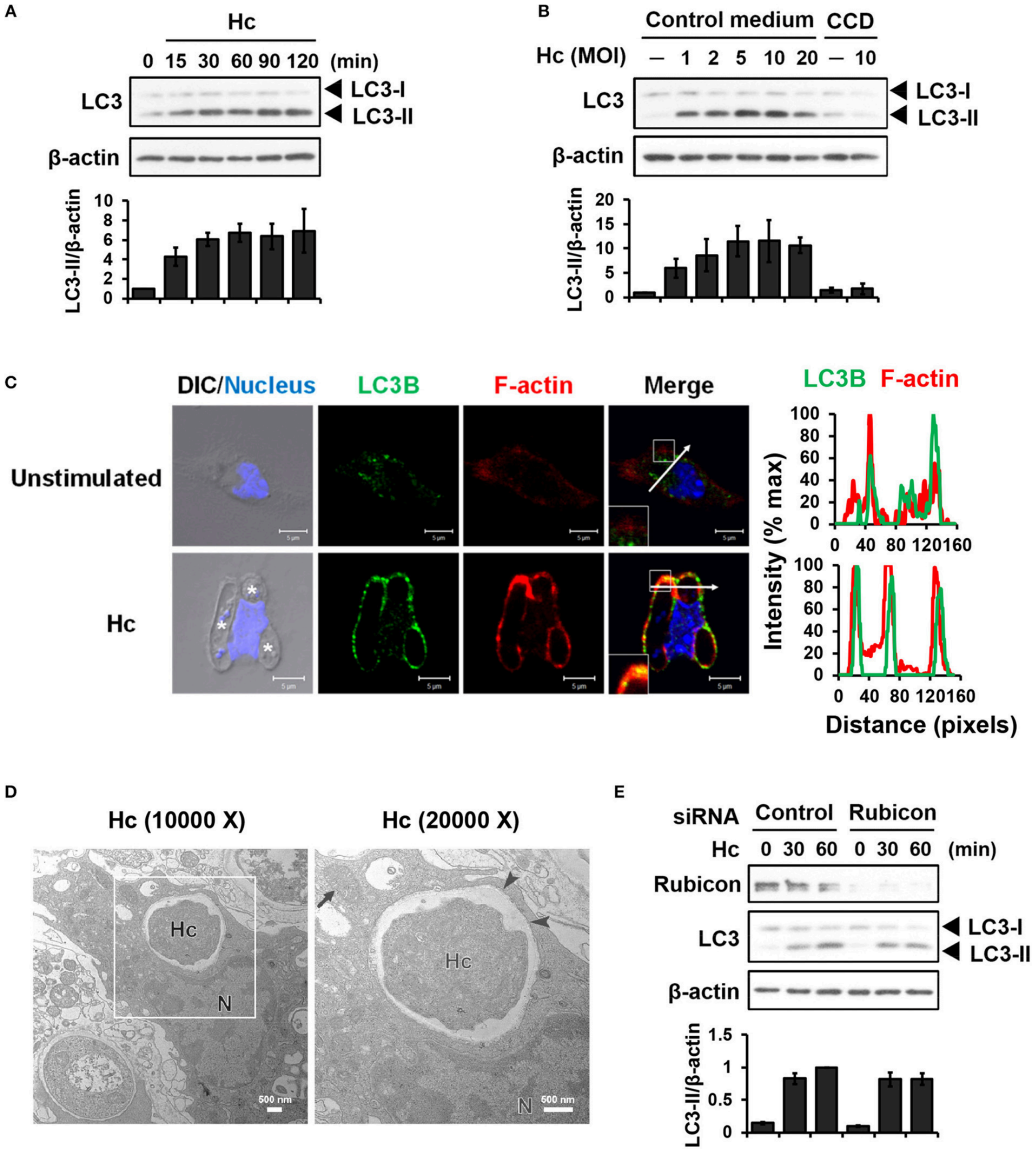
Infection of macrophages with *H. capsulatum* induces LC3 conversion and recruitment to fungus-containing phagosome. **(A)** Macrophages from WT mice were stimulated with or without (0 min) *H. capsulatum* (MOI = 5) for the indicated time. Cell lysates were extracted and analyzed by Western blotting. **(B)** Macrophages from WT mice treated with or without cytochalasin D (CCD, 10 μg/ml) were stimulated with *H. capsulatum* at the indicated yeast-to-macrophage ratios for 60 min. Cell lysates were extracted and analyzed by Western blotting. **(C)** Macrophages were stimulated with or without *H. capsulatum* (MOI = 5) for 60 min. Cells were fixed and stained for LC3B (green), F-actin (red), and nucleus compartment (blue). Cells were viewed under confocal microscope. Asterisks in the DIC/Nucleus field point to *H. capsulatum* yeasts. Box areas are shown at higher magnification in the bottom left corner of the corresponding image. The intensity of different fluorochromes along the white arrow in the merged image is shown as the histogram on the right. Data shown are representative of at least 3 independent experiments with similar results. **(D)** Macrophages from WT mice were stimulated with or without *H. capsulatum* (MOI = 2) for 30 min. Transmission electron microscopy (TEM) were used to analyze *H. capsulatum*-containing vacuoles in macrophages. Representative TEM micrographs are shown. Box area in the middle panel is shown as magnified image in the right panel. **(E)** Macrophages from WT mice were transfected with control siRNA or siRNA against Rubicon (50 nM) for 72 h. Cells were then stimulated with or without (0 min) *H. capsulatum* (MOI = 5) for 30 and 60 min. After stimulation, cell lysates were collected and assessed by Western blotting. Data shown in the lower panel are relative intensity of LC3-II normalized against the corresponding β-actin, mean ± SEM are shown (*n* = 3) **(A,B,E)**.

LC3-associated phagocytosis (LAP) is characterized by conjugation of lipidated LC3 to a single phagosomal membrane following receptor-mediated recognition of microbial particles (15). We sought to determine whether *H. capsulatum* induces LAP in primary mouse peritoneal macrophages. Western blot analysis showed that *H. capsulatum* stimulation induced LC3-II (the lipidated form of LC3) formation within 15 min and peaked at 60 min after infection (Figure 1A). While increasing multiplicity of infection (MOI, up to 10) increased the levels of LC3-II, cytochalasin D treatment, which inhibits phagocytosis, abolished LC3-II formation (Figure 1B). Confocal images revealed that LC3 was diffusely distributed in unstimulated macrophages (Figure 1C). Its intensity increased and localized to phagosomes upon macrophage uptake of the fungus (Figure 1C). Furthermore, *H. capsulatum*-containing vesicle was a single- but not a double-membrane structure (Figure 1D). Yet, while silencing Rubicon by small interfering RNA (siRNA) inhibited zymosan-induced LC3 conversion (Figures S1A, S2), it did not affect *H. capsulatum*-induced LC3-II formation (Figure 1E). These observations provide evidence that LAPosome is formed in macrophages after engulfing *H. capsulatum*. While Rubicon is required for LAP upon uptake of zymosan (23), it is dispensable for *H. capsulatum*-induced LAP.

### *H. capsulatum* induces LAP through dectin-1

**Figure 2:**
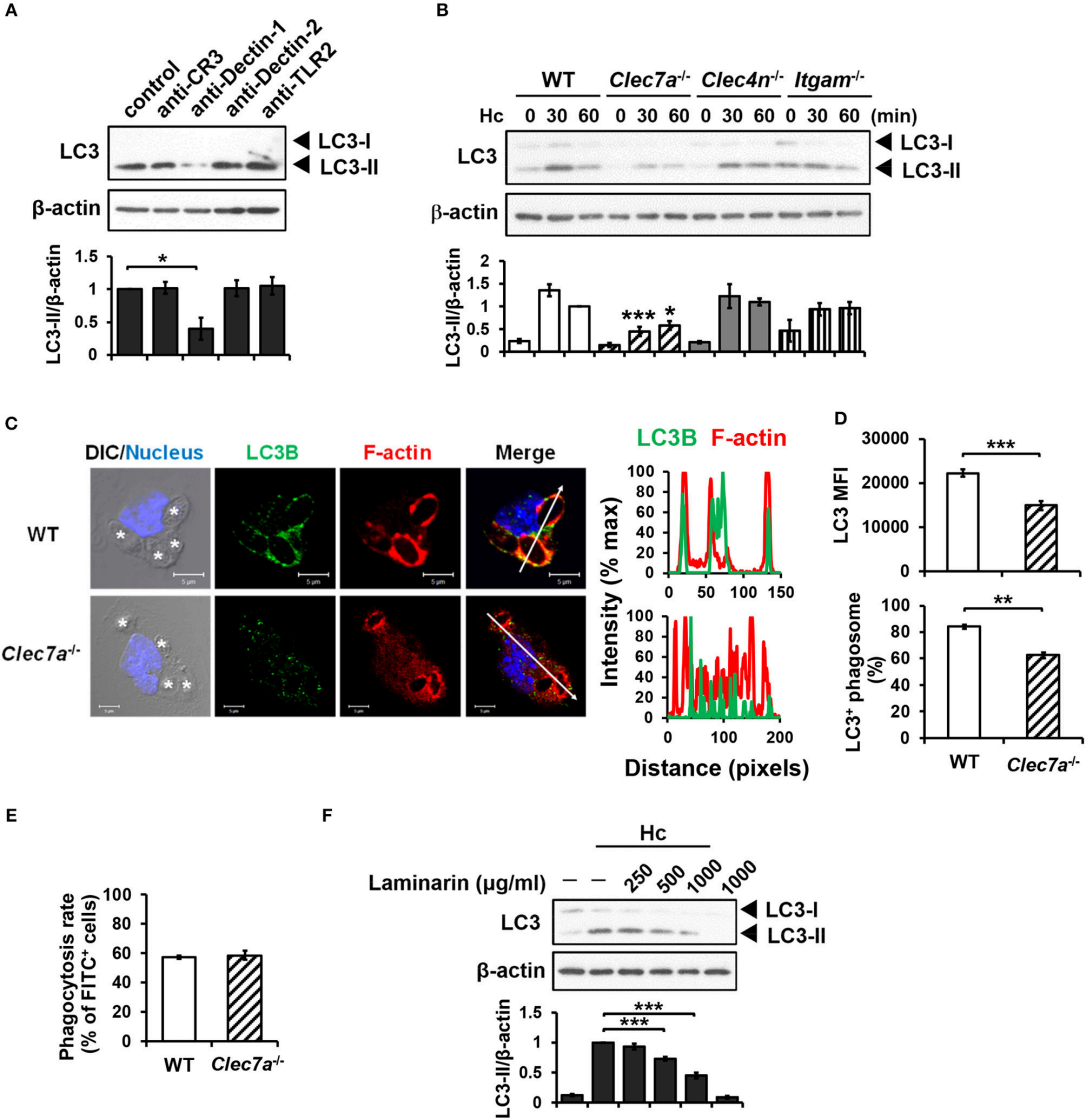
Dectin-1-mediated recognition is crucial for *H. capsulatum-*induced LC3-II formation. **(A,F)** Macrophages from WT mice were treated with blocking antibodies against CR3, Dectin-1, Dectin-2, or TLR2 (10 μg/ml each) **(A)** or laminarin at indicated concentrations **(F)** for 1 h prior to stimulation with *H. capsulatum* (MOI = 5) for 1 h. Cell lysates were extracted and analyzed by Western blotting. **(B)** Macrophages from WT, *Clec7a*^−/−^, *Clec4n*^−/−^ and *Itgam*^−/−^ mice were stimulated with or without (0 min) *H. capsulatum* (MOI = 5) for 30 and 60 min. Cell lysates were subjected to Western blotting. Data shown in the lower panel are relative intensity of LC3-II normalized against the corresponding β-actin, mean ± SEM are shown [*n* = 3 for **(A,B)**, *n* = 5 for **(F)**]. **(C,D)** Macrophages from WT and *Clec7a*^−/−^ mice were stimulated with *H. capsulatum* (MOI = 5) for 1 h. Cells were fixed and stained for LC3B (green), F-actin (red), and the nucleus compartment (blue). Cells were viewed under confocal microscope. Asterisks in the DIC/Nucleus image point to *H. capsulatum* yeasts. The intensity of different fluorochromes along the white arrow in the merged image is shown as the histogram on the right. The mean fluorescence intensity (MFI) of LC3 in cells engulfing *H. capsulatum* was quantified. Phagosomes in each cell were counted and the percentages of LC3^+^ phagosome are shown. Bars represent the mean ± SEM of 3 independent experiments. **(E)** Macrophages from WT and *Clec7a*^−/−^ mice were allowed to phagocytose FITC-labeled *H. capsulatum* (MOI = 10) for 1 h. Percentages of cells taking up *H. capsulatum* were analyzed by flow cytometry. Mean ± SEM are shown (*n* = 4). *p ≤ 0.05, **p ≤ 0.01, ***p ≤ 0.001 [ANOVA with Bonferroni's multiple comparisons *post-hoc* test **(A,B,F)**; 2-tailed *t*-test **(D,E)**].

Blocking antibodies and receptor-deficient cells were employed to determine the receptor(s) that is involved in LAP induction in *H. capsulatum*-infected macrophages. Blockade of Dectin-1 but not CR3, Dectin-2 or TLR2 inhibited LC3-II formation (Figure 2A). Consistently, LC3 conversion was reduced in *H. capsulatum*-stimulated Dectin-1-deficient (*Clec7a*^−/−^) macrophages, whereas the level of LC3-II in macrophages deficient in Dectin-2 (*Clec4n*^−/−^) or CR3 (*Itgam*^−/−^) was comparable to cells from WT mice (Figure 2B). Immunofluorescence images also showed that the intensity of endogenous LC3 and the percentage of LC3-positive phagosomes were lower in *Clec7a*^−/−^ macrophages (Figures 2C,D), yet Dectin-1 deficiency did not affect phagocytosis of the organism (Figure 2E). Treatment with laminarin, a soluble β-glucan, dose-dependently blocked *H. capsulatum*-induced LC3-II formation (Figure 2F). These data demonstrate that Dectin-1 is critical to macrophage recognition of β-glucan exposed on the surface of *H. capsulatum* for LAP induction.

### NADPH oxidase-mediated ROS production is involved in *H. capsulatum*-induced LAP

**Figure 3:**
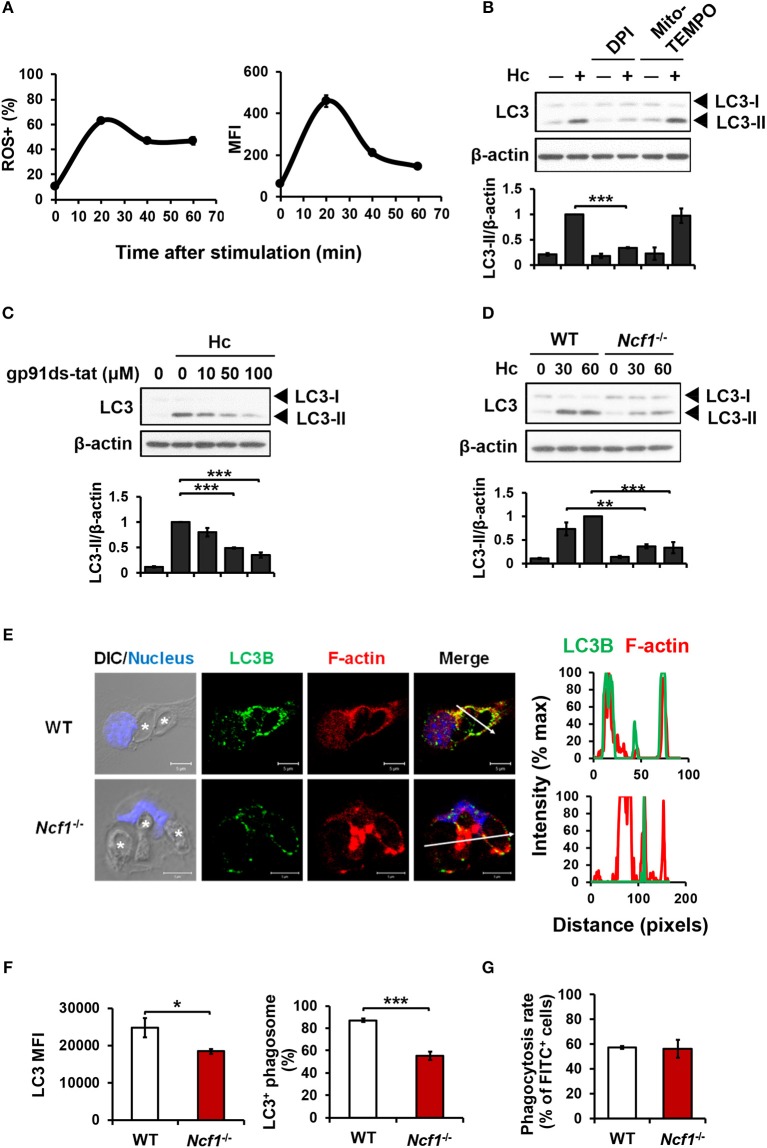
NADPH oxidase-mediated ROS response to *H. capsulatum* is essential for LC3 conversion in macrophages. **(A)** Macrophages from WT mice were preloaded with CM-H_2_DCFDA for 30 min prior to stimulation with *H. capsulatum* (MOI = 10) for indicated time. Flow cytometry was performed to assess global ROS production. Data shown are the percentages of ROS^+^ cells (left panel) and the mean fluorescence intensity (MFI) (right panel) of total live cells. Mean ± SEM are shown (*n* = 4). **(B,C)** Macrophages from WT mice were treated with or without DPI (5 μM), Mito-Tempo (10 μM) **(B)**, and gp91ds-tat (10, 50, and 100 μM) **(C)** for 1 h prior to stimulation with *H. capsulatum* (MOI = 5). Cell lysates were collected after 1 h of stimulation and analyzed by Western blotting. **(D)** Macrophages from WT or *Ncf1*^−/−^ mice were stimulated with or without (0 min) *H. capsulatum* (MOI = 5) for 30 and 60 min. Cell lysates were subjected to Western blotting. Data shown in the lower panel are relative intensity of LC3-II normalized against the corresponding β-actin; mean ± SEM are shown. [*n* = 3 for **(B,C)**; *n* = 4 for **(D)**]. **(E,F)** Macrophages from WT and *Ncf1*^−/−^ mice were stimulated with *H. capsulatum* (MOI = 5) for 1 h. Cells were fixed and stained for LC3B (green), F-actin (red), and the nucleus compartment (blue). Cells were viewed under confocal microscope. Asterisks in the DIC/Nucleus image point to *H. capsulatum* yeasts. The intensity of different fluorochromes along the white arrow in the merged image is shown as histogram on the right. The mean fluorescence intensity (MFI) of LC3 in cells engulfing *H. capsulatum* was quantified. Phagosomes in each cell were counted and the percentages of LC3^+^ phagosome are shown as mean ± SEM of 4 independent experiments. **(G)** Macrophages from WT and *Ncf1*^−/−^ mice were allowed to phagocytose FITC-labeled *H. capsulatum* (MOI = 10) for 1 h. Percentages of cells taking up *H. capsulatum* were analyzed by flow cytometry. Mean ± SEM are shown (*n* = 4). *p ≤ 0.05, **p ≤ 0.01, ***p ≤ 0.001 [ANOVA with Tukey *post-hoc* analysis **(B,C)** or Bonferroni's multiple comparisons *post-hoc* test **(D)**; 2-tailed *t*-test **(F,G)**].

ROS involvement in *H. capsulatum*-induced LAP was examined. We found that while *H. capsulatum* induced ROS production (Figure 3A), diphenyleneiodonium (DPI) but not Mito-TEMPO treatment reduced *H. capsulatum*-induced LC3-II formation (Figure 3B). In addition, treatment with gp91ds-tat, a peptide inhibitor of NADPH oxidase assembly or p47^phox^ deficiency (*Ncf1*^−/−^) separately reduced LC3-II conversion (Figures 3C,D). Fluorescence imaging further showed that lacking p47^phox^ attenuated LC3 intensity and localization to the phagosomes and reduced the percentage of LC3-positive phagosomes (Figures 3E,F), while p47^phox^ deficiency did not affect the ability of macrophage to phagocytose the organism (Figure 3G). These results demonstrate that NADPH oxidase-mediated ROS response is required for LC3 conversion in macrophage response to *H. capsulatum*.

### Syk acts downstream of dectin-1 for LAP induction via regulating NADPH oxidase-mediated ROS response

**Figure 4:**
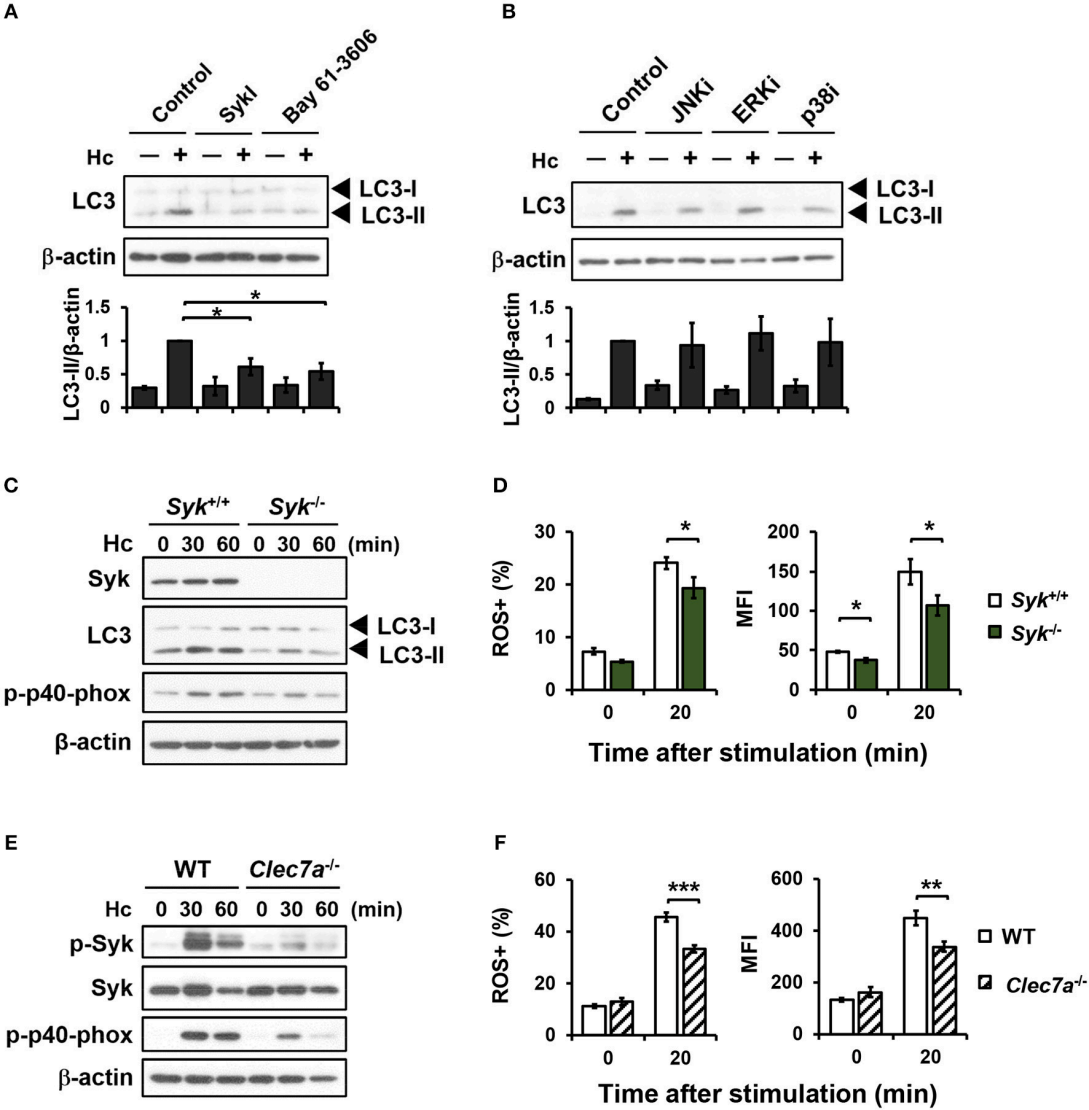
Syk acts downstream of Dectin-1 and affects LC3-II formation by regulating ROS production. **(A,B)** Macrophages from WT mice were treated with Syk inhibitors (SyKI, 10 μM; Bay 61-3606; 3 μM) **(A)** and MAPK inhibitors [SP600125 (JNKi, 20 μM); U0126 (ERKi, 20 μM); SB203580 (p38i, 20 μM)] **(B)** for 45 min prior to stimulation with *H. capsulatum* (MOI = 5) for 1 h. After stimulation, cell lysates were collected and assessed by Western blotting. Data shown in the lower panel are relative intensity of LC3-II normalized against the corresponding β-actin (*n* = 3). **(C,E)** FLDMs from *Syk*^+/+^ (WT) and *Syk*^−/−^ embryos **(C)** or macrophages from WT and *Clec7a*^−/−^ mice **(E)** were stimulated with or without (0 min) *H. capsulatum* (MOI = 5) for 30 and 60 min. After stimulation, cell lysates were collected and assessed by Western blotting for the expression of indicated proteins. **(D,F)** FLDMs from *Syk*^+/+^ and *Syk*^−/−^ embryos **(D)** or macrophages from WT and *Clec7a*^−/−^ mice **(F)** were preloaded with CM-H_2_DCFDA for 30 min prior to stimulation with or without (0 min) *H. capsulatum* (MOI = 10) for 20 min. Flow cytometry was performed to assess global ROS production. Data shown are the percentages of ROS^+^ cells (left panel) and the mean fluorescence intensity (MFI) (right panel) of total live cells. [*n* = 3–4 **(D)** and *n* = 6 **(F)**]. Bars represent the mean ± SEM. *p ≤ 0.05, **p ≤ 0.01, ***p ≤ 0.001 [ANOVA with Tukey *post-hoc* analysis **(A,B)** or Bonferroni's multiple comparisons *post-hoc* test **(D,F)**].

Dectin-1 engagement is known to activate downstream Syk and MAPKs (7, 39). Pharmacological inhibition of Syk significantly reduced *H. capsulatum*-induced LC3-II formation (Figure 4A), while inhibiting JNK, ERK, and p38 had no effect on LC3 conversion (Figure 4B). Syk or Dectin-1 deficiency separately reduced macrophage p40^phox^ phosphorylation, ROS production, and LC3-II formation upon encountering *H. capsulatum* (Figures 4C–F). These results together show that Dectin-1/Syk mediates LC3 conversion in macrophage response to *H. capsulatum* by regulating NADPH oxidase-mediated ROS production.

### NLRX1 facilitates *H. capsulatum*-induced LAP

**Figure 5:**
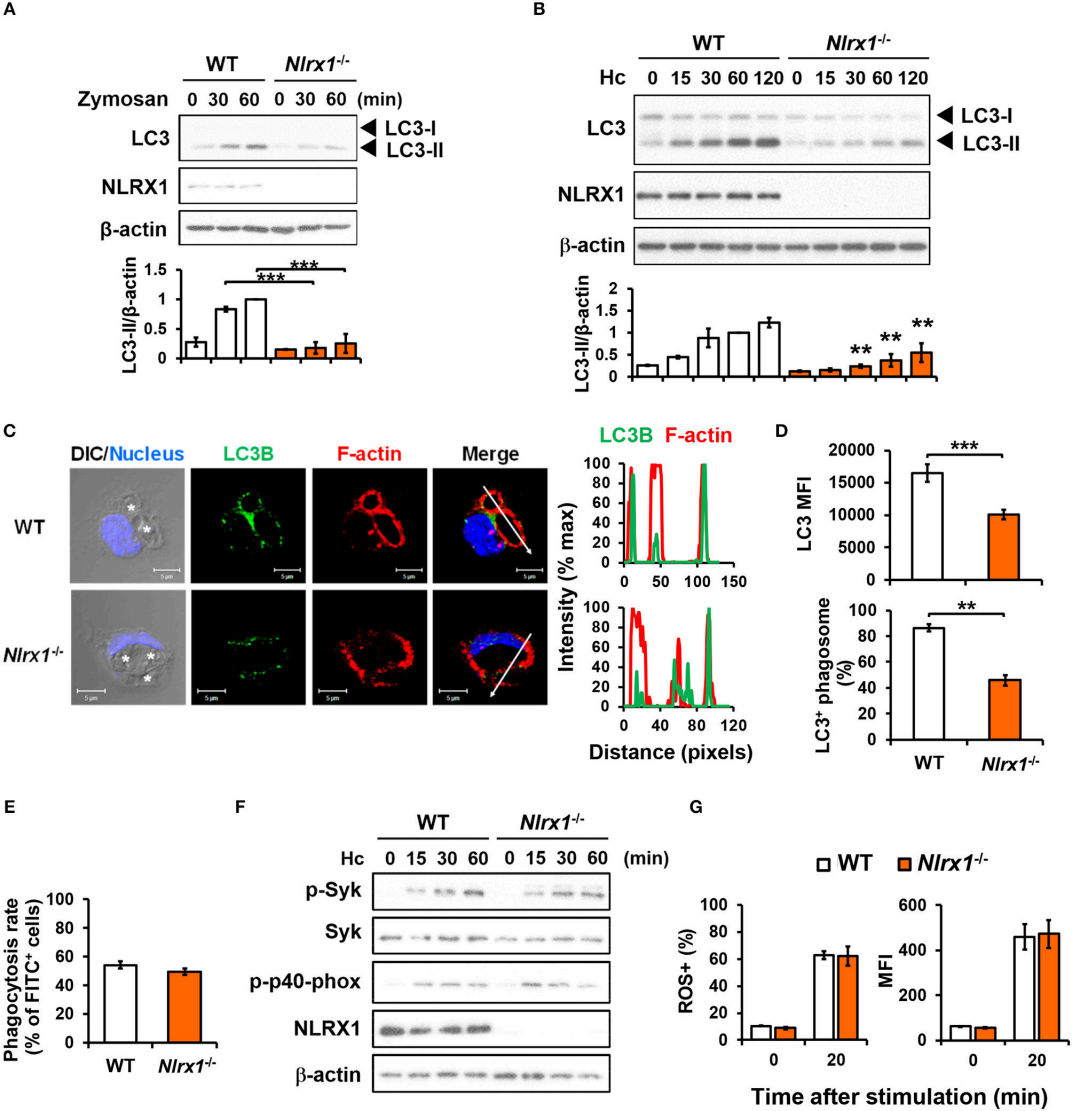
NLRX1 acts independently of ROS response to promote *H. capsulatum*-induced LC3 conversion in macrophages. **(A,B,F)** Macrophages from WT and *Nlrx1*^−/−^ mice were stimulated with or without (0 min) zymosan (50 μg/ml) **(A)** or *H. capsulatum* (MOI = 5) **(B,F)** for indicated time. Cell lysates were extracted and analyzed by Western blotting for the expression of indicated proteins. Data shown in the lower panel are relative intensity of LC3-II normalized against the corresponding β-actin, mean ± SEM are shown (*n* = 3) **(A,B)**. **(C,D)** Macrophages from WT and *Nlrx1*^−/−^ mice were stimulated with *H. capsulatum* for 1 h. Cell were fixed and stained for LC3B (green), F-actin (red), and nucleus compartment (blue), and viewed under confocal microscope **(C)**. Asterisks in the DIC/Nucleus field point to *H. capsulatum* yeasts. The intensity of different fluorochromes along the white arrow in the merged image is shown as the histogram on the right. The mean fluorescence intensity (MFI) of LC3 in cells engulfing *H. capsulatum* was quantified. Phagosomes in each cell were counted and the percentages of LC3^+^ phagosome are shown as mean ± SEM of 3 independent experiments **(D)**. **(E)** Macrophages from WT and *Nlrx1*^−/−^ mice were allowed to phagocytose FITC-labeled *H. capsulatum* (MOI = 10) for 1 h. Percentages of cells engulfing *H. capsulatum* were analyzed by flow cytometry (*n* = 7). **(G)** Macrophages from WT and *Nlrx1*^−/−^ mice were preloaded with CM-H_2_DCFDA for 30 min prior to stimulation with *H. capsulatum* (MOI = 10) for 20 min. Flow cytometry was performed to assess global ROS production. Data shown are the percentages of ROS^+^ cells (left panel) and the mean fluorescence intensity (MFI) (right panel) of total live cells (*n* = 4). Bars represent the mean ± SEM. **p ≤ 0.01, ***p ≤ 0.001 [ANOVA with Bonferroni's multiple comparisons *post-hoc* test **(A,B, G)**; 2-tailed *t*-test **(D,E)**].

We employed macrophages from *Nlrx1*^−/−^ mice to investigate whether NLRX1 plays a role in fungus-induced LAP. Results in Figures 5A,B show that *Nlrx1* deficiency significantly reduced LC3-II formation in macrophages after stimulation by both zymosan and *H. capsulatum*. Immunofluorescence imaging also showed that the intensity of endogenous LC3 and the percentage of LC3-positive phagosomes were significantly reduced in *Nlrx1*^−/−^ macrophages (Figures 5C,D), yet reduction in LC3 conversion in *Nlrx1*^−/−^ macrophages was not the result of reduced phagocytosis of the fungus (Figure 5E). It is worth noting that NLRX1 was distributed in cytoplasm as well as in the mitochondria of unstimulated macrophages (Figure S3). Upon uptake of *H. capsulatum*, NLRX1 translocated to fungus-containing phagosomes (Figure S3). Furthermore, *Nlrx1* deficiency did not affect Syk and p40^phox^ phosphorylation (Figure 5F), nor did it affect the percentage and the intensity of ROS-positive cells (Figure 5G). Thus, NLRX1 acts independently of Syk and ROS response to promote *H. capsulatum*-induced LC3 conversion.

### NLRX1 interacts with TUFM and cooperates with ROS-dependent pathway for LAP induction

**Figure 6:**
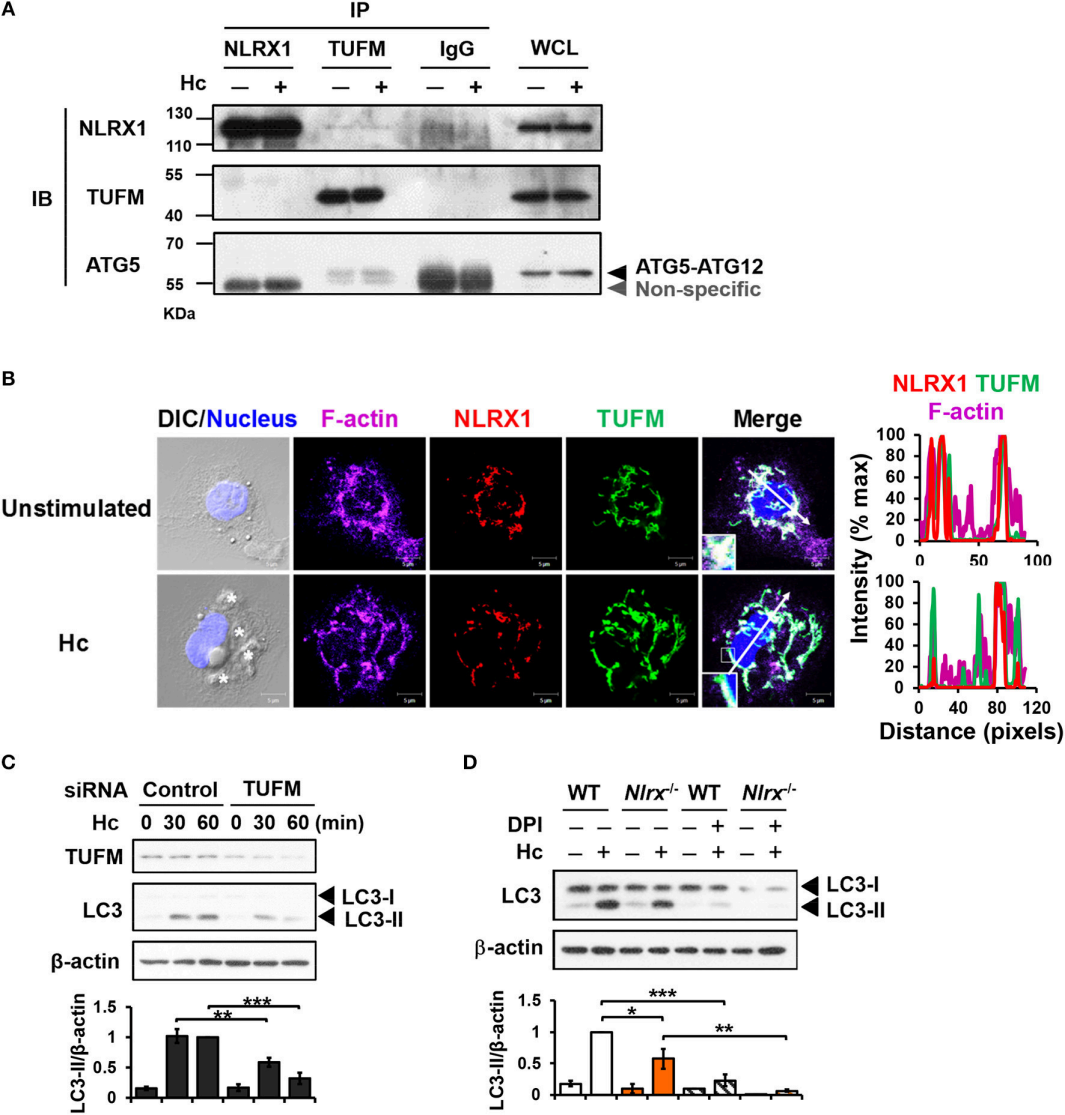
NLRX1 promotes LAP through association with TUFM-ATG5-ATG12 complex. **(A)** Macrophages from WT mice were stimulated with or without *H. capsulatum*. Cell lysates were collected at 30 min after stimulation and used for immunoprecipitation with anti-NLRX1, anti-TUFM or isotype control antibodies, followed by immunoblotting with indicated antibodies. IP, IB, and WCL denote immunoprecipitation, immunoblotting, and whole-cell lysate, respectively. **(B)** Macrophages were stimulated with or without *H. capsulatum* (MOI = 5) for 60 min. Cells were fixed and stained for NLRX1 (red), TUFM (green), F-actin (violet), and nucleus compartment (blue). Cells were viewed under confocal microscope. Asterisks in the DIC/Nucleus field point to *H. capsulatum* yeasts. Box areas are shown at higher magnification in the bottom left corner of the corresponding image. The intensity of different fluorochromes along the white arrow in the merged image is shown as histogram on the right. **(C)** Macrophages from WT mice were transfected with control siRNA or siRNA against TUFM (50 nM) for 72 h. Cells were then stimulated with or without (0 min) *H. capsulatum* (MOI = 5) for 30 and 60 min. After stimulation, cell lysates were collected and assessed by Western blotting. **(D)** Macrophages from WT and *Nlrx1*^−/−^ mice were treated with or without DPI (5 μM) for 1 h prior to stimulation with *H. capsulatum* (MOI = 5). Cell lysates were collected after 1 h of stimulation and analyzed by Western blotting. Data shown in the lower panel of **(C,D)** are relative intensity of LC3-II normalized against the corresponding β-actin, mean ± SEM are shown (*n* = 3). *p ≤ 0.05, **p ≤ 0.01, ***p ≤ 0.001 [ANOVA with Bonferroni's multiple comparisons *post-hoc* test **(C,D)**].

Previous work showed that NLRX1 and TUFM promote autophagy (29). To explore their roles in LAP, co-immunoprecipitation experiments were performed to determine whether NLRX1 interacts with TUFM to facilitate *H. capsulatum*-induced LC3-II formation. Results showed that endogenous TUFM co-immunoprecipitated with endogenous NLRX1 and ATG5-ATG12 conjugate in macrophages with or without *H. capsulatum* stimulation (Figure 6A). Immunofluorescence images also demonstrated that a part of TUFM colocalized with NLRX1, regardless of fungal stimulation (Figure 6B). Silencing TUFM by siRNA significantly reduced LC3-II formation in macrophages at 30 and 60 min after encountering *H. capsulatum* (Figure S1B and Figure 6C). These data show that NLRX1 associates with TUFM-ATG5-ATG12 complex and that possibly by this association NLRX1 promotes *H. capsulatum*-induced LAP. Furthermore, LC3-II induction was further reduced in *Nlrx1*^−/^^−^ macrophages treated with DPI compared to DPI-untreated *Nlrx1*^−/^^−^ and DPI-treated NLRX1-sufficient macrophages (Figure 6D), indicating that NLRX1-TUFM complex-dependent pathway and Dectin-1-mediated ROS-dependent pathway collaboratively contribute to LAP induction.

### LAP induction by viable *H. capsulatum* does not lead to phagosomal acidification

**Figure 7:**
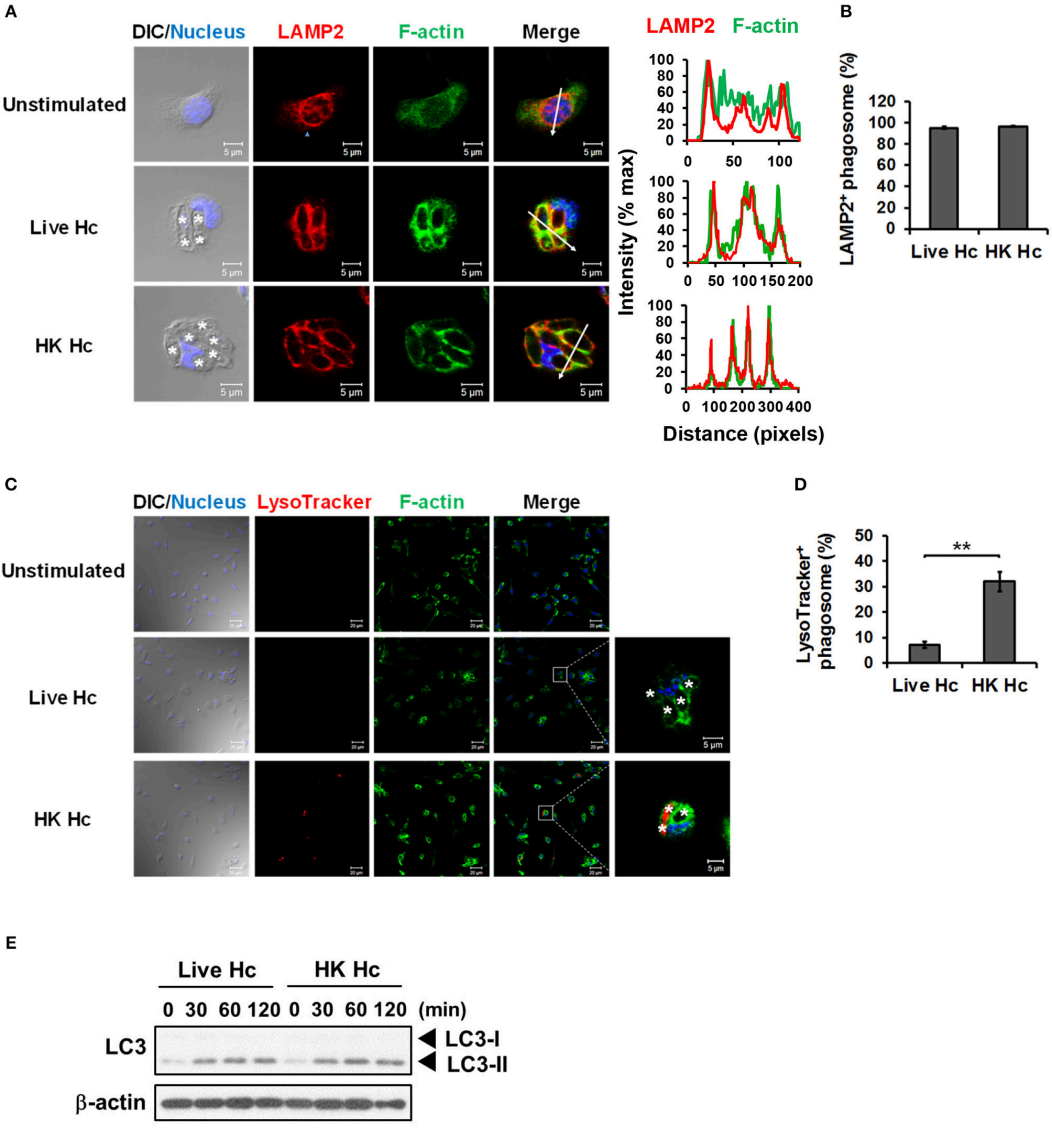
Viable *H. capsulatum* prevents phagosomal acidification in macrophages. **(A,B)** Macrophages from WT mice were stimulated with or without live or heat-killed (HK) *H. capsulatum* (MOI = 5) for 4 h. Cells were fixed and stained for LAMP2 (red), F-actin (green), and nucleus compartment (blue). Cells were viewed under confocal microscope. Asterisks in the DIC/Nucleus field point to *H. capsulatum* yeasts. The intensity of different fluorochromes along the white arrow in the merged image is shown as the histogram on the right. Phagosomes in each cell were counted and the percentages of LAMP2^+^ phagosome in cells taking up live and heat-killed fungus are shown as mean ± SEM in bar graphs (*n* = 3) **(B)**. **(C,D)** Macrophages from WT mice were stimulated with or without live or heat-killed (HK) *H. capsulatum* (MOI = 5) for 2 h and unengulfed yeasts were washed off. Macrophages were then culture in 100 nM LysoTracker Red for additional 2 h to detect phagolysosomal acidification. Cell were fixed and stained for F-actin (green) and nucleus compartment (blue). Cells were viewed under confocal microscope. Box areas are shown at higher magnification in the left of the corresponding image and asterisks in the magnified field point to *H. capsulatum* yeasts. Data shown are representative of 3 independent experiments with similar results **(A,C)**. Phagosomes in each cell were counted and the percentages of LysoTracker^+^ phagosome in cells taking up live and heat-killed *H. capsulatum* are shown. **(E)** Macrophages from WT mice were stimulated with or without (0 min) live or heat-killed (HK) *H. capsulatum* (MOI = 5) for 30, 60, 90, and 120 min. Cell lysates were extracted and analyzed by Western blotting. Bars represent the mean ± SEM (*n* = 4) **(D)**. **p ≤ 0.01 [2-tailed *t*-test **(B,D)**].

It is reported that LAP promotes phagosome maturation that leads to degradation of internalized materials (15, 23, 40). We examined whether phagolysosomal fusion and its acidification occurs to *H. capsulatum*-containing phagosomes. Results showed that while LAMP2 localized to phagosomes containing either heat-killed or viable *H. capsulatum* (Figures 7A,B), acidification only occurred to phagosomes containing heat-killed but not to those containing viable organisms (Figures 7C,D). Consistent with what has been reported, these data indicate that phagosome-lysosome fusion occurs in macrophages engulfing of either heat-killed or viable *H. capsulatum*, and that viable organisms are capable of actively preventing phagosome acidification (5). It is of interest to note that both viable and heat-killed yeasts induced LC3-II formation at a comparable level (Figure 7E). Together these results indicate that phagosome acidification is not a natural consequence of LAP.

### LAP formation promotes MAPKs-AP-1 activation and cytokine response in *H. capsulatum*-infected macrophages

**Figure 8:**
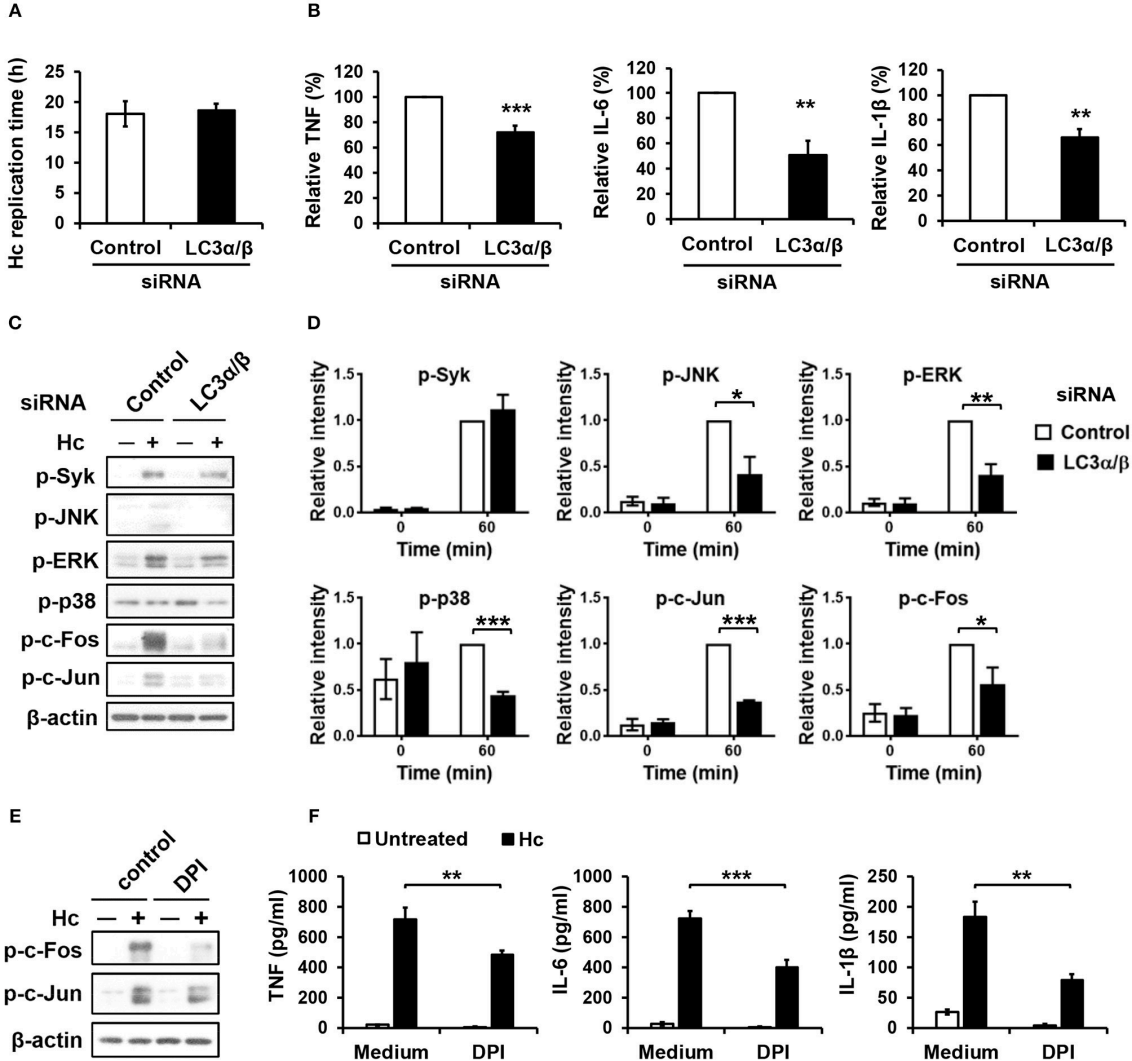
LC3 is required for activation of MAPKs-AP-1 pathway and cytokine production upon *H. capsulatum* stimulation. **(A–D)** Macrophages from WT mice were transfected with control siRNA or siRNA against LC3α/β (25 nM) for 72 h before stimulation with *H. capsulatum* [MOI = 2 **(A)** and 5 **(B,C)**]. For **(A)**, macrophages were washed to rid of unengulfed yeasts at 1 h after stimulation then lysed immediately (0 h) or after 18 h of incubation, and the number of yeast cells were counted. Replication time (h) = incubation interval/number of divisions (*n* = 5). For **(B)**, culture supernatants were collected at 18 h after stimulation, and the concentrations of TNF, IL-6, and IL-β in the supernatants were quantified by ELISA and are presented as the relative levels of each cytokine (*n* = 6 for TNF and IL-6; *n* = 4 for IL-1β). For **(C)**, cell lysates were collected at 1 h after stimulation and analyzed by Western blotting for the expression of indicated proteins. Relative intensity of indicated protein normalized against the corresponding β-actin was shown in **(D)** (*n* = 3). **(E,F)** Macrophages from WT mice were treated with or without DPI (5 μM) for 1 h prior to stimulation with *H. capsulatum* (MOI = 5). Cell lysates were collected at 1 h after stimulation and analyzed by Western blotting **(E)**. Culture supernatants were collected at 18 h after stimulation, and the concentrations of TNF, IL-6, and IL-β in the supernatants were quantified by ELISA (*n* = 4) **(F)**. Bars represent the mean ± SEM, *p ≤ 0.05, **p ≤ 0.01, ***p ≤ 0.001 [2-tailed *t*-test **(A,B, D)**; ANOVA with Bonferroni's multiple comparisons *post-hoc* test **(F)**].

Next, we sought to assess the role of LC3 conversion in macrophage interaction with *H. capsulatum*. We found that silencing LC3α/β did not affect intracellular replication of *H. capsulatum* but caused reduced TNF, IL-6, and IL-1β production (Figures S1C, Figures 8A,B). While Syk phosphorylation was not affected, silencing LC3α/β reduced the levels of phosphorylated JNK, ERK, p38, c-Fos, and c-Jun (AP-1) (Figures 8C,D). Interestingly, inhibition of ROS response by DPI suppressed AP-1 activation and downregulated TNF, IL-6, and IL-1β response to *H. capsulatum* (Figures 8E,F). These results together with those in Figure 3 demonstrate that ROS-mediated LC3 conversion is required for optimal MAPKs-AP-1 activation and proinflammatory cytokine production in macrophage response to *H. capsulatum* infection.

### NLRX1-TUFM complex positively regulates macrophage cytokine response to *H. capsulatum* by enhancing MAPKs-AP-1 activation

**Figure 9:**
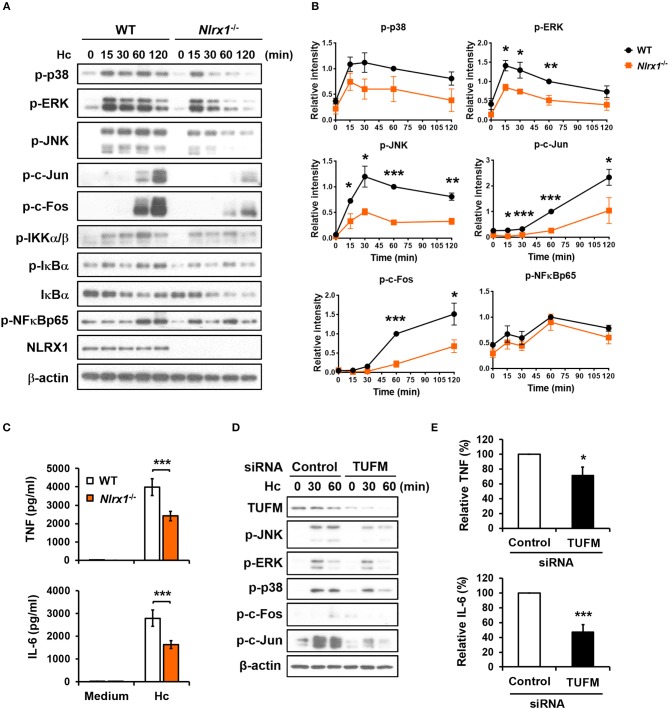
NLRX1-TUFM is required for activation of MAPKs-AP-1 pathway and proinflammatory cytokine response to *H. capsulatum*. **(A–C)** Macrophages from WT and *Nlrx1*^−/−^ mice were stimulated with or without (0 min or medium) *H. capsulatum* (MOI = 5). Cell lysates were collected at 15, 30, 60, and 120 min after stimulation and subjected to Western blotting for the analysis of the indicated proteins **(A)**. Relative intensity of indicated protein normalized against the corresponding β-actin was shown in **(B)** (*n* = 3). Supernatants were harvested at 18 h after stimulation, and the concentrations of TNF and IL-6 in the supernatants were quantified by ELISA (*n* = 11) **(C)**. **(D,E)** Macrophages from WT mice were transfected with control siRNA or siRNA against TUFM (50 nM) for 72 h. Cells were then stimulated with or without (0 min) *H. capsulatum* (MOI = 5). Cell lysates were collected at 30 and 60 min after stimulation and assessed by Western blotting for the analysis of the indicated proteins **(D)**. Supernatants were harvested at 18 h after stimulation, and the concentrations of TNF and IL-6 in the supernatants were quantified by ELISA and are presented as the relative levels of TNF and IL-6 (*n* = 6) **(E)**. Bars represent the mean ± SEM, *p ≤ 0.05, **p ≤ 0.01, ***p ≤ 0.001 [2-tailed *t*-test **(B,E)**; ANOVA with Bonferroni's multiple comparisons *post-hoc* test **(C)**].

Given that NLRX1 is involved in *H. capsulatum*-induced LAP, we next explored whether NLRX1 modulates macrophage signaling activation and anti-fungal proinflammatory cytokine response. Western blot analysis showed that in contrast to what was observed in LPS stimulation (Figure S4) (27, 28), *Nlrx1* deficiency reduced the phosphorylation of p38, ERK, JNK, c-Fos, and c-Jun (AP-1) but did not affect the signaling molecules in the NF-κB pathway upon stimulation by *H. capsulatum* (Figures 9A,B). The production of TNF and IL-6 was also significantly reduced in *Nlrx1*^−/−^ macrophages (Figure 9C). Interestingly, silencing TUFM reduced MAPKs-AP-1 activation as well as TNF and IL-6 production (Figures 9D,E, and Figure S5). These data together with our previous finding that AP-1 activation is crucial to macrophage cytokine response (7) demonstrate that NLRX1-TUFM complex positively regulates macrophage cytokine response to *H. capsulatum* by promoting MAPKs-AP-1 activation.

## Discussion

LC3-associated phagocytosis (LAP) bridging PRR signaling to autophagic machinery has been described as an innate defense mechanism against fungal pathogens (20–24). Recruitment of LC3 to single-membrane phagosomes and the requirement of NADPH oxidase-mediated ROS for LC3 recruitment and conversion are hallmarks of LAP (15, 41). In this study, we showed that *H. capsulatum* is targeted by LAP in mouse peritoneal macrophages. LC3 is readily converted to LC3-II and translocates to *H. capsulatum*-containing single-membrane vesicles. LC3-II formation is diminished when NADPH oxidase-mediated ROS response is inhibited. Unlike what is reported with zymosan-induced LAP, Rubicon is not involved in *H. capsulatum*-induced non-canonical autophagy. By genetic approaches and use of specific pharmacological inhibitors, we also delineated the underlying molecular mechanism and the biological functions of LAP in *H. capsulatum*-infected macrophages. Furthermore, we identified novel roles for NLRX1 and TUFM in fungus-induced LAP.

Our results demonstrated that targeting *H. capsulatum* by LAP is mediated by Dectin-1, but not CR3, Dectin-2, or TLR2. Signal downstream of Dectin-1 activates Syk and thereby triggers NADPH oxidase-mediated ROS production that is essential for LAP induction. It is interesting to note that while CR3 is responsible for both macrophage phagocytosis and cytokine response to *H. capsulatum* (7, 38), it is not required for LAP induction. We observed that *H. capsulatum*-induced p40^phox^ phosphorylation was not affected by CR3 deficiency (Figure S6). CR3 not being involved in NADPH oxidase-mediated ROS production could be the explanation for why it does not mediate LAP induction. Our finding that Dectin-1 deficiency does not completely abolish LC3 conversion and translocation to phagosomes suggests that Dectin-1 may not be the only receptor involved in LAP induction. Which receptor(s) other than Dectin-1 is involved in LAP induction still awaits to be identified.

It has been shown that LAP can modulate inflammatory cytokine response to fungi. Macrophages deficient in LC3β produce higher levels of TNF and IL-1β after stimulation with *C. albicans* (22). A different report showed that LC3β deficiency does not affect Dectin-1-mediated TNF and IL-6 responses to fungal antigens by DCs (19). Silencing ATG5 enhances macrophage IL-6 but reduces IP-10 response to *C. neoformans* (20). Mice deficient in components involved in LAP pathway exhibit enhanced inflammation and inflammatory cytokine response after infection with *A. fumigatus* (23, 42). However, DCs defective in LAP have reduced inflammatory cytokine response to commensal yeasts, *Saccharomyces cerevisiae* and *Kazachstania unispora* (43). We showed that abrogating LAP by silencing LC3α/β or inhibiting ROS production impairs TNF, IL-6, and IL-1β response to *H. capsulatum* through interfering MAPKs-AP-1 activation. It appears that whether LAP enhances or mitigates inflammatory cytokine response depends on the type of fungal stimulant and the type of host cells. In addition, autophagy and inflammasome complex are known to intersect and regulate each other to maintain cellular and immune homeostasis (44). However, the role of LAP in regulation of inflammasome is still largely unclear. It has been reported that IFN-γ-induced LAP promotes NLRP3 proteasomal degradation upon *A. fumigatus* infection via DAPK1 (42). Inhibiting DAPK1 results in enhanced inflammasome activation and IL-1β/IL-18 production, likely due to impaired LAP induction (42). We recently revealed the importance of NLRP3 inflammasome-mediated IL-1β response in host defense against *H. capsulatum* (45). In contrast to *A. fumigatus* infection, our results suggest that LAP positively regulates *H. capsulatum*-induced inflammasome activation. Since activation of NLRP3 inflammasome by *H. capsulatum* is mediated by Syk-JNK pathway (45), it is likely that LAP-mediated JNK activation acts upstream of NLRP3 inflammasome for IL-1β production. *C. albicans*-induced autophagy is important to the activation of NF-κB by sequestering its inhibitor, A20 (46). It is possible that some negative regulators of MAPKs may be targeted by autophagic machinery that is activated by *H. capsulatum*. Further studies are needed to elucidate how LAP regulates the activation of MAPKs-AP-1 pathway and the interplay between LAP and inflammasome.

Phagosome maturation is a process involving a series of steps including phagosome-lysosome fusion and phagolysosome acidification, ultimately leading to degradation of internalized particles. LAP is believed to facilitate phagosome maturation, as macrophages defective in LAP are impaired in degrading internalized *S. cerevisiae* and *A. fumigatus* (15, 23). Here we showed that silencing LC3α/β does not affect intracellular replication of *H. capsulatum*. Our results along with previous studies demonstrate that viable, but not heat-killed, *H. capsulatum* prevents phagolysosome acidification and escapes from killing in macrophages (5, 6). Although the precise mechanism utilized by *H. capsulatum* for intracellular survival is still unclear, a study of mutant strains revealed that 3-hydroxy-3-methylglutaryl coenzyme A lyase (HCL1) involved in leucine catabolism is critical for maintaining neutral pH in phagolysosomes (47). The *hcl1* mutation results in accumulation of acidic intermediates in the leucine catabolic pathway which acidify *H. capsulatum*-containing phagosome, thereby compromises the growth of *H. capsulatum* in macrophages (47). Our results showing no difference in the levels of LAP and cytokine induction by viable or heat-killed organisms (7) indicate that LAP is involved in macrophage cytokine response but not phagosome maturation during *H. capsulatum* infection. It is likely that specific features of a pathogen and its interaction with host cells could influence the biological outcomes of LAP.

It has been shown that NLRX1 promotes VSV-induced autophagy by forming a complex with TUFM which in turn associates with autophagic proteins, ATG5-ATG12 and ATG16L1 (29). However, the role of NLRX1 in non-canonical autophagy has not been demonstrated. By employing macrophages from *Nlrx1*^−/−^ mice, we demonstrated that NLRX1 is required for LAP induction by zymosan and *H. capsulatum*. NLRX1 interacts with TUFM which is associated with ATG5-ATG12 conjugate. Lacking NLRX1 or silencing TUFM markedly reduces LC3 conversion, indicating the formation of NLRX1-TUFM complex is essential for LAP induction. Apart from being involved in microbial infection, LAP is required for efficient clearance of dead cells (40). It is recently reported that by preventing inflammation during clearance of dead cells, LAP plays a key role in suppressing autoimmune response and promoting tumor growth (48, 49). Mice lacking any of the components involved in LAP pathway in myeloid cells exhibit reduced capacity to clear dying cells and develop systemic lupus erythematosus (SLE)-like syndromes accompanied by elevated inflammatory cytokines and autoantibodies (48). Another study showed that tumor-associated macrophages lacking LAP promote anti-tumor T cell activity through a mechanism dependent on STING-mediated type I IFN response (49). Given the importance of NLRX1 in LAP and inflammation, our findings raise the possibility that NLRX1 may be implicated in the development of autoimmune disease and tumor by regulating LAP induction.

NLRX1 is known to both positively and negatively regulate cytokine production. It plays a negative role in inflammatory cytokine and type I IFN production through modulating the signaling cascades activated by TLR stimulation and viral infection (25, 27–29, 32, 50). Through dynamic interactions with TRAF6 and IKK complex, NLRX1 attenuates NF-κB activation which leads to downregulation of TNF, IL-6, IL-1β, and IFN-β production in LPS-stimulated macrophages (27, 28). On the other hand, direct association of NLRX1 with MAVS and STING inhibits MAVS-RLR pathway and STING-TBK1 signaling, respectively, thereby suppresses type I IFN response and promotes viral infection (25, 27, 32, 50). NLRX1 exerts positive effects on innate antiviral immunity (51, 52). *Nlrx1*^−/−^ macrophages exhibit impaired type I IFN response to influenza A virus infection (51). Sendai virus-induced IRF1 signaling is potentiated by NLRX1 through increasing IRF1 expression in hepatocytes (52). Our results showed that NLRX1 positively regulates macrophage cytokine response. *Nlrx1* deficiency results in impairment of LAP through which attenuates MAPKs-AP-1 signaling and cytokine response to *H. capsulatum*. It is demonstrated that NLRX1 localization can dynamically change after stimulation (53). It is our speculation that NLRX1 localization to different subcellular compartments after stimulation by different stimuli may determine its role in cellular response.

**Figure 10:**
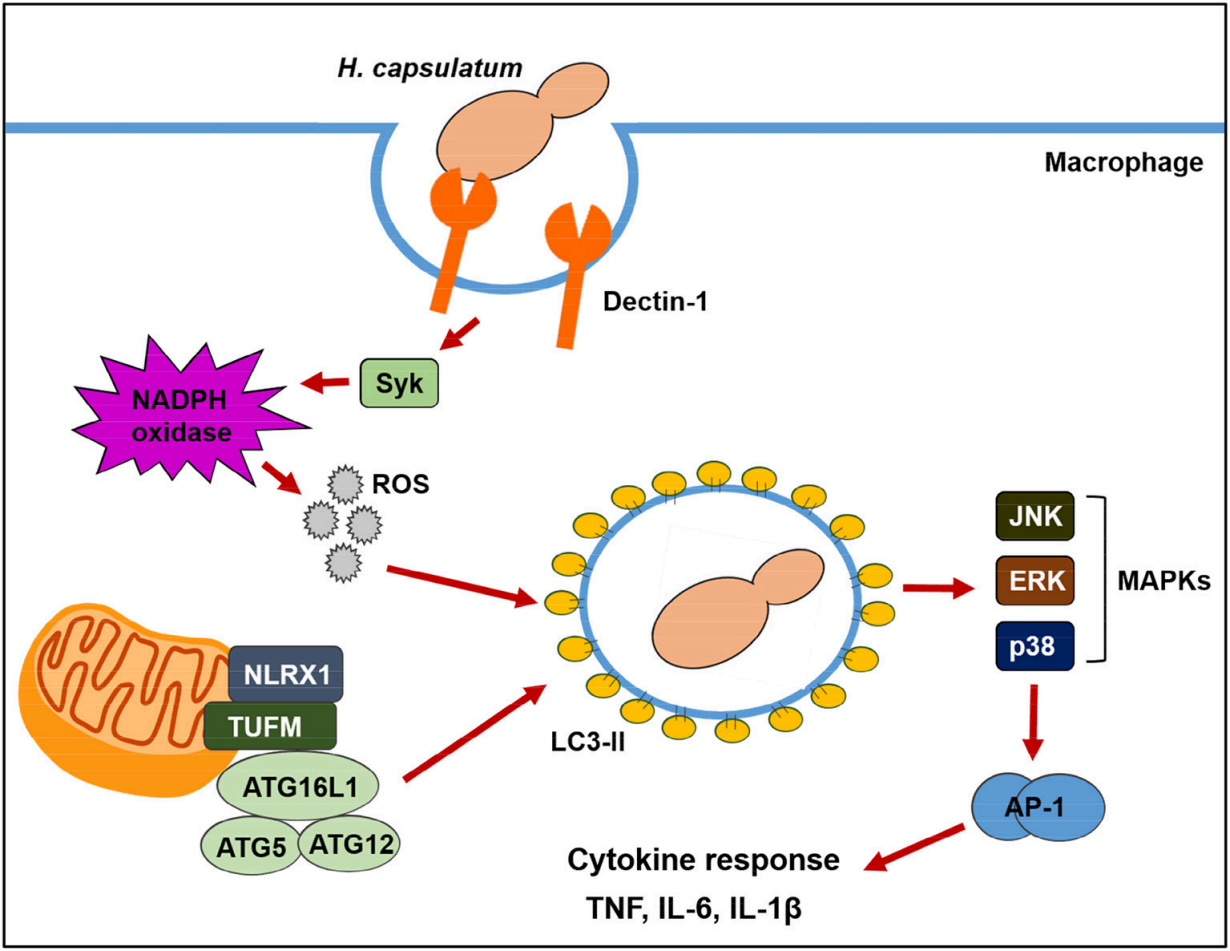
Schematic model of LAP in *H. capsulatum*-infected macrophage. Upon macrophage encountering *H. capsulatum*, recognition of fungal β-glucan by Dectin-1 induces Syk phosphorylation. Activation of Syk triggers ROS production through NADPH oxidase. ROS is required for converting LC3-I to LC3-II on phagosomal membrane. Through a mechanism independent of ROS, NLRX1 promotes fungus-induced LAP through its association with TUFM that interacts with ATG5-ATG12 conjugate for LAPosome formation. Both Dectin-1/Syk/ROS-dependent pathway and NLRX1-TUFM complex-dependent pathway collaboratively contribute to LAP induction. The formation of LAPosome promotes downstream MAPKs and AP-1 activation, leading to production of anti-fungal proinflammatory cytokines.

Based on our observations, we proposed a model as illustrated in Figure 10 for the two pathways contributing to LAP in macrophage response to *H. capsulatum* infection. Upon engagement with β-1,3-glucan on *H. capsulatum*, Dectin-1 activates Syk and triggers NADPH oxidase-derived ROS production, resulting in LAP. NLRX1 facilitates LAP induction independent of NADPH oxidase by forming a complex with an intermediary partner, TUFM, which is associated with autophagic proteins, ATG5-ATG12 and ATG16L1. LAP promotes the activation of MAPKs-AP-1 pathway which is essential for proinflammatory cytokine production. Our findings provide a connection between NLRX1 and LAP and show that both ROS-dependent and ROS-independent pathways drive anti-fungal cytokine response in macrophages.

## Author contributions

J-HH and BW-H conceived and designed experiments. J-HH, C-YL, S-YW, W-YC, and T-HC performed experiments and analyzed data. H-WK and S-TH provided technical assistance and performed experiments for transmission electron microscopy analysis. JPT edited the article and provided technical assistance and materials for NLRX1 study. J-HH, S-YW, and BW-H drafted and finalized the manuscript. J-HH and BW-H supervised and coordinated the work.

### Conflict of interest statement

The authors declare that the research was conducted in the absence of any commercial or financial relationships that could be construed as a potential conflict of interest.
