# Supplementary material for: NLRX1 Facilitates Histoplasma capsulatum-Induced LC3-Associated Phagocytosis for Cytokine Production in Macrophages
Source: Front Immunol. 2018 Dec 3;9:2761. doi: 10.3389/fimmu.2018.02761 (PMC6286976; doi:10.3389/fimmu.2018.02761)
Supplement: Supplementary file 1 [file Data_Sheet_1.docx]

***Supplementary Materials***

**NLRX1 Facilitates *Histoplasma capsulatum*-Induced LC3-Associated Phagocytosis for Cytokine Production in Macrophages**

**Juin-Hua Huang, Chu-Yu Liu, Sheng-Yang Wu, Wen-Yu Chen, Tzu-Hsuan Chang, Hung-Wei Kan, Sung-Tsang Hsieh, Jenny P.-Y. Ting, Betty A. Wu-Hsieh^*^**

*** Correspondence:** Betty A. Wu-Hsieh: [bwh@ntu.edu.tw](mailto:bwh@ntu.edu.tw)

**
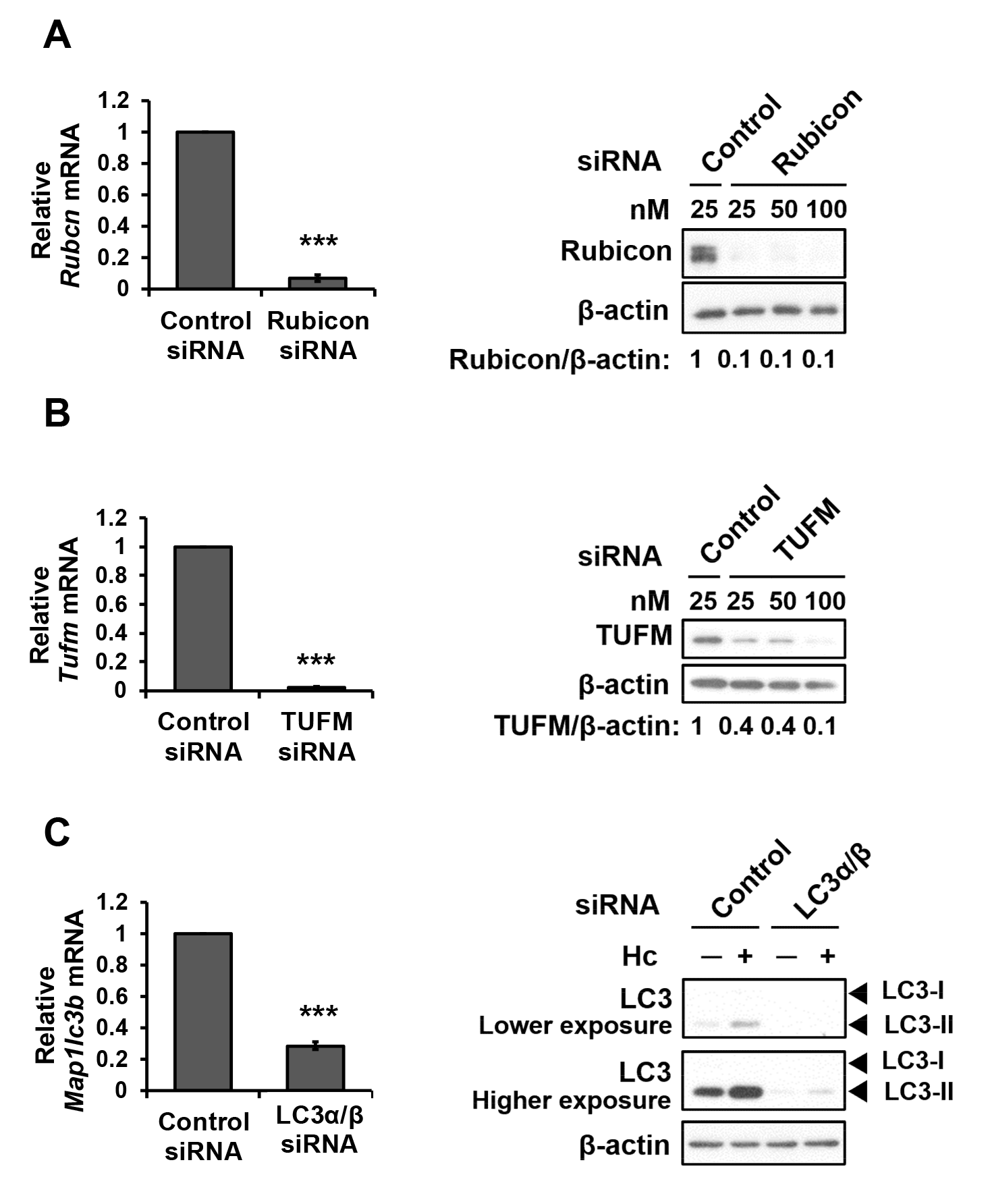
**

**Figure S1. Silencing Rubicon, TUFM, and LC3α/β by siRNA.** Macrophages from WT mice were transfected with control siRNA or siRNA against Rubicon (A), TUFM (B), and LC3α/β (C). After transfection for 30 h (left panels) and 72 h (right panels), total mRNA (left panels) and cell lysates (right panels) were extracted. The expression levels of indicated transcripts and proteins were determined by quantitative RT-PCR (left panels) and Western blotting (right panels), respectively. Transcript and protein of β-actin were used as an internal control. Bars represent the mean ± SEM [n = 3 (A and C); n = 4 (B)]. *** *p* ≤ 0.001 (2-tailed *t*-test).


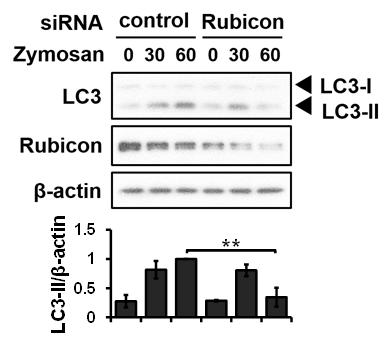


**Figure S2. Rubicon is involved in zymosan-induced LAP.** Macrophages from WT mice were transfected with control siRNA or siRNA against Rubicon (50 nM) for 72 h. Cells were then stimulated with or without (0 min) zymosan (50 μg/ml) for 30 and 60 min. After stimulation, cell lysates were collected and assessed by Western blotting for the expression of indicated proteins. Data shown in the lower panel are relative intensity of LC3-II normalized against the corresponding β-actin, mean ± SEM are shown (n = 3). ** *p* ≤ 0.01, (ANOVA with Bonferroni’s multiple comparisons post-hoc test).


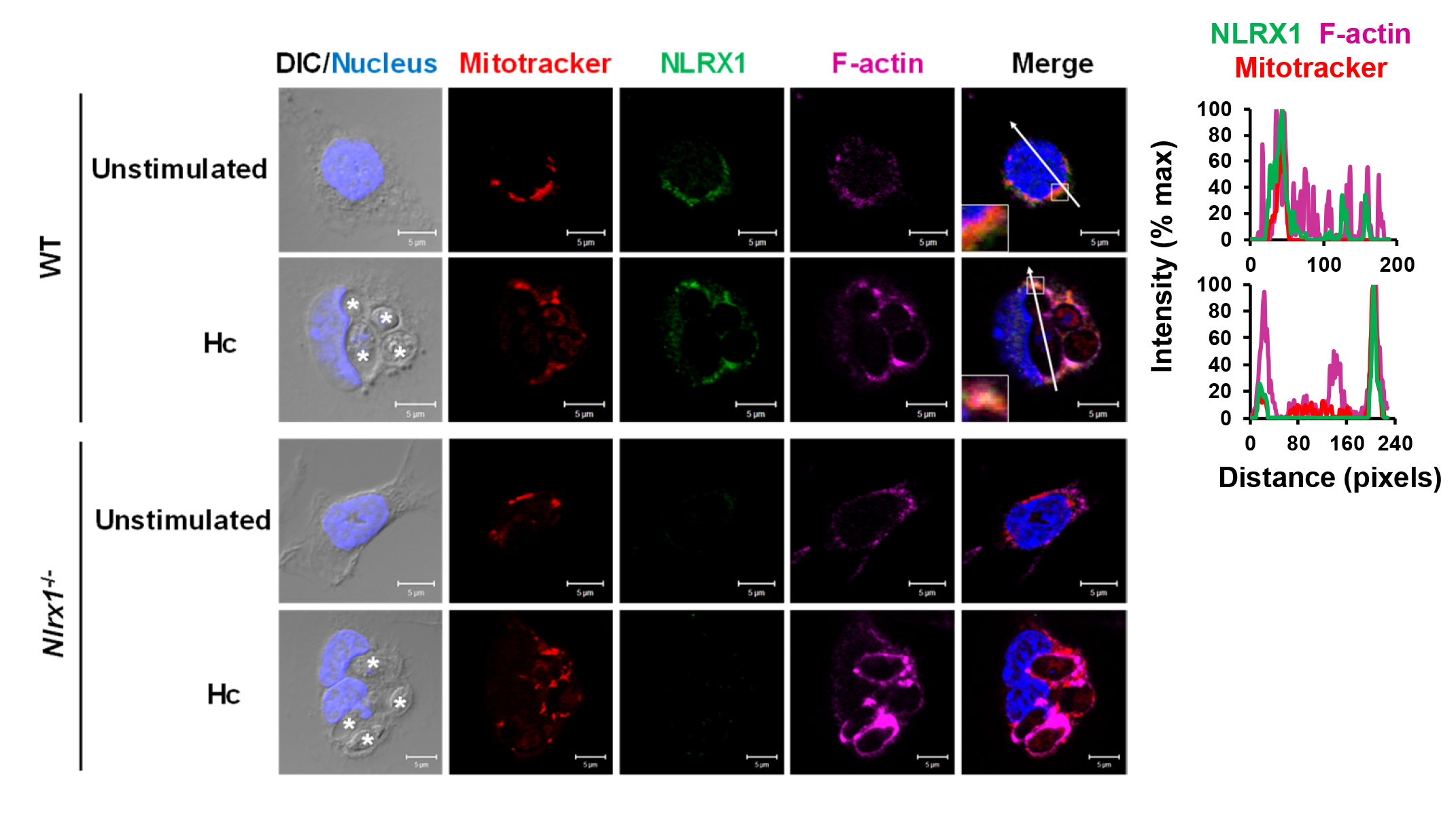


**Figure S3. NLRX1 is recruited to phagosome in *H. capsulatum*-infected macrophages.** Macrophages from WT and *Nlrx1*^-/-^ mice were loaded with Mitotracker Red (red) and stimulated with or without *H. capsulatum* for 1 h. Cells were fixed and stained for NLRX1 (green), F-actin (violet), and nucleus compartment (blue) and viewed under confocal microscope. Asterisks in the DIC/Nucleus field point to *H. capsulatum* yeasts. *Nlrx1*^-/-^ cells with or without *H. capsulatum* stimulation were used as negative controls for staining by primary antibodies. Box areas are shown at higher magnification in the bottom left corner of the corresponding image. The intensity of different fluorochromes along the white arrow in the merged image is shown as the histogram on the right. Data shown are representative of at least 3 independent experiments with similar results.


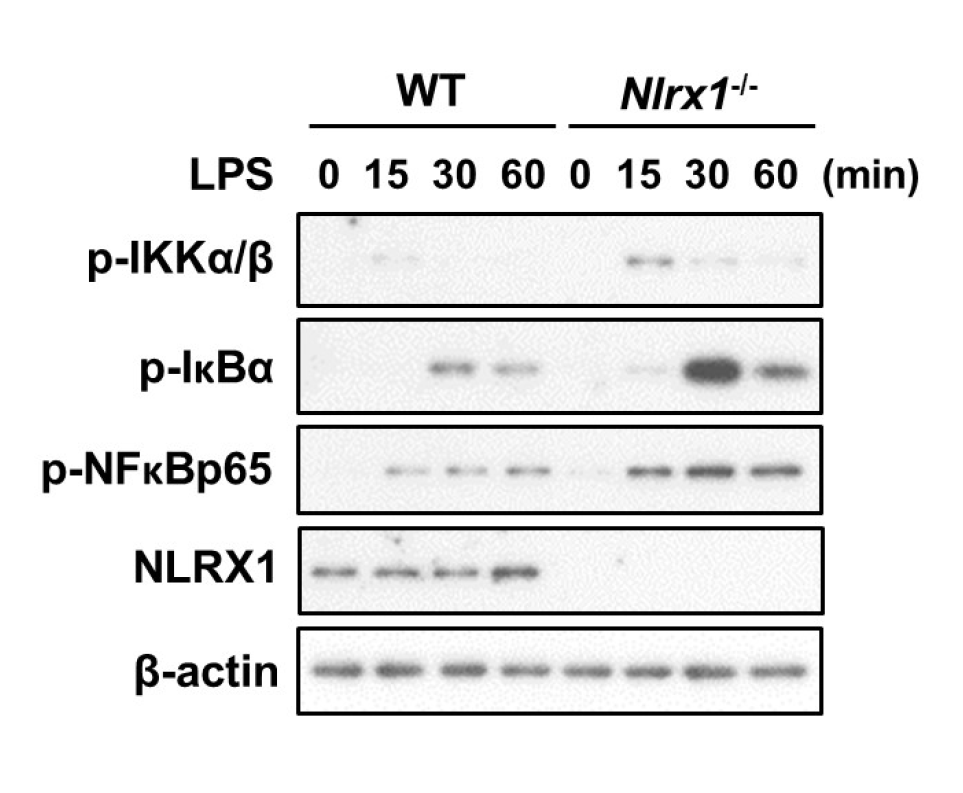


**Figure S4. NLRX1 negatively regulates NF-κB activation in LPS-stimulated macrophages.** Macrophages from WT and *Nlrx1*^-/-^ mice were stimulated with or without (0 min) LPS (1 μg/ml) for 15, 30, and 60 min. Cell lysates were extracted and analyzed by Western blotting for p-IKKα/β, p-IκBα, p-NF-κBp65 and NLRX1 expression. Beta-actin was used as a loading control.

**
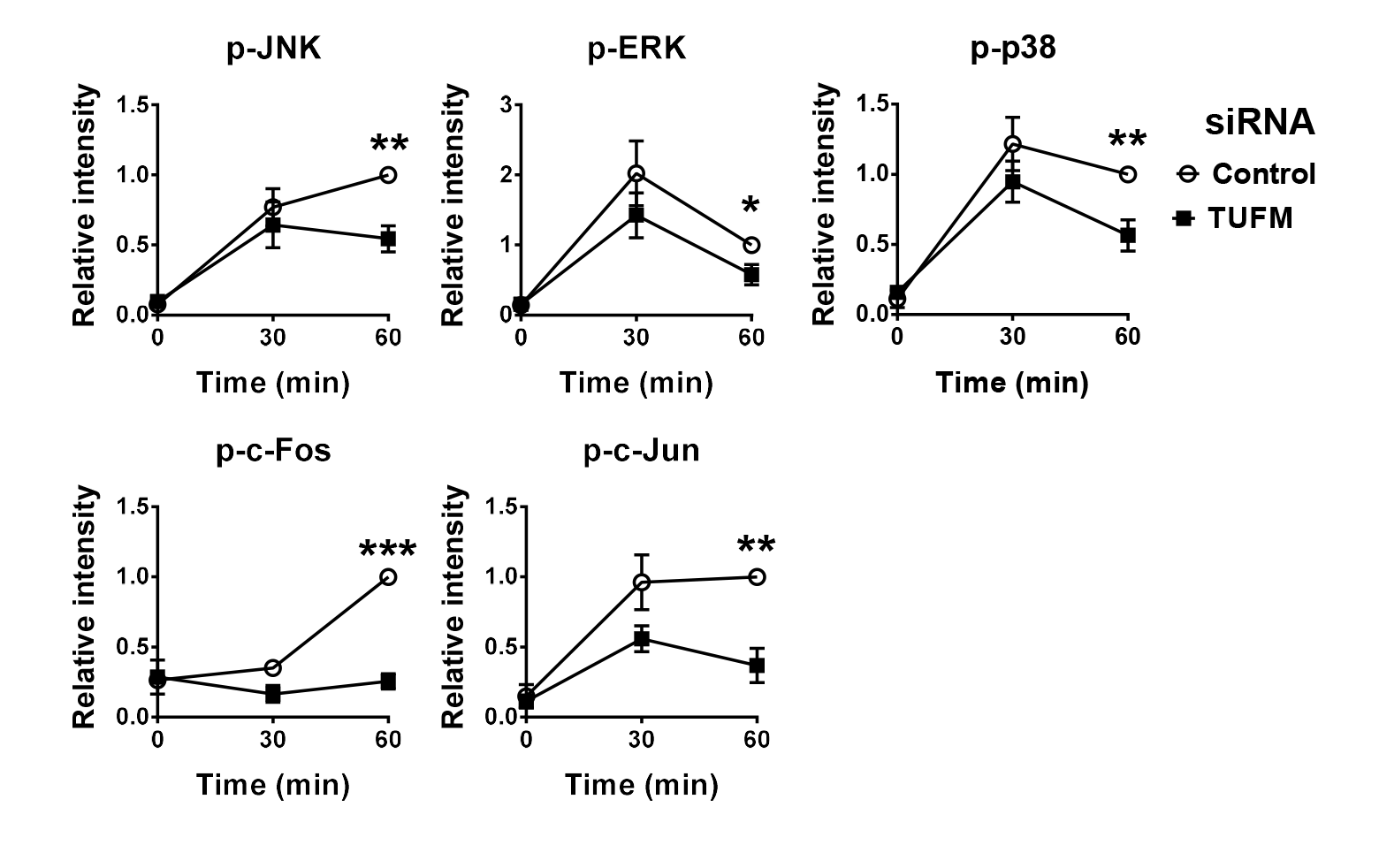
**

**Figure S5. Silencing TUFM reduced MAPK-AP-1 signaling activated by *H. capsulatum*.** Macrophages from WT mice were transfected with control siRNA or siRNA against TUFM (50 nM) for 72 h. Cells were then stimulated with or without (0 min) *H. capsulatum* (MOI = 5). Cell lysates were collected at 30 and 60 min after stimulation and analyzed for MAPKs and AP-1 signaling molecules by Western blotting. One representative of three independent experiments is presented in Fig 9D. Relative intensity of indicated phosphorylated molecules were quantified by ImageJ. Error bars indicate SEM (n =3), * *p* ≤ 0.05, ** *p* ≤ 0.01, *** *p* ≤ 0.001 (2-tailed *t*-test).


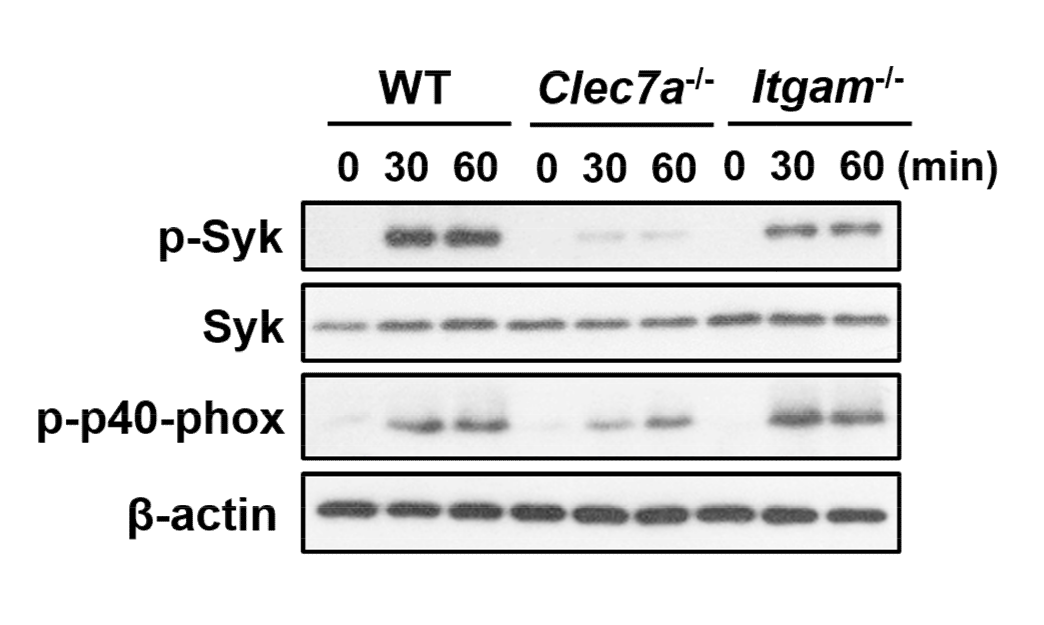


**Figure S6. CR3 deficiency does not affect *H. caspsulatum*-induced p40-phox phosphorylation.** Macrophages from WT, *Clec7a*^-/-^, *Itgam*^-/-^ mice were stimulated with or without (0 min) *H. capsulatum* (MOI = 5) for 30 and 60 min. After stimulation, cell lysates were collected and assessed by Western blotting for the expression of indicated proteins.

**Supplementary method**

**Real-time qPCR**

Total RNA was extracted from macrophages using RNAzol RT (Molecular Research Center) according to the manufacturer’s instructions. cDNA was reversely transcribed in reaction mixture containing extracted RNA, first-strand buffer, DTT, dNTP, oligo dT (Invitrogen), SuperScript III reverse transcriptase (Invitrogen) and RNaseOUT recombinant ribonuclease inhibitor (Invitrogen). For real-time qPCR, cDNA was mixed with forward and reversed primers in Fast STBR Green Master Mix (Applied Biosystems). The reaction was performed with a PikoReal software 96 Real-Time PCR system (Thermo Scientific), and the results were analyzed by PikoReal software 2.1. The primers used for real-time qPCR included: *Rubcn* (Rubicon): 5’-TCA GGG TGT AGT GCA TGG TT-3’ (forward) and 5’- GAC GCA TCC GGA CTA TAA GG-3’ (reverse); *Tufm* (TUFM): 5’- GCA GCC ACT CTA TTG CGA G-3’ (forward) and 5’- CCG ACC TTG CAG AAA TGG G-3’; *Map1lc3b* (LC3B): 5’- GAC GGC TTC CTG TAC ATG GTT-3’ (forward) and 5’- TGG AGT CTT ACA CAG CCA TTG-3’ (reverse); *Actb* (β-actin) 5’-TGT ATG AAG GCT TTG GTC TCC CT-3’ (forward) and 5’-AGG TGT GCA CTT TTA TTG GTC TCA A-3’ (reverse).
